# Effects of Photoperiods on Growth Performance, Serum Biochemical Parameters, Slaughter Performance, Skeletal Development, and Meat Quality of Yellow-Feathered Broilers

**DOI:** 10.3390/ani16142240

**Published:** 2026-07-19

**Authors:** Jiasheng Li, Ziying Rong, Shuangchun Zhang, Chunyan Luo, Xiaohang Nie, Yong Chen, Jiancheng Liu

**Affiliations:** Research Center for Biological Feed and Animal Gut Health, College of Animal Science, Xinjiang Agricultural University, Urumqi 830052, China; ljs1693095665@163.com (J.L.); 15066028862@163.com (Z.R.); 15739968269@163.com (S.Z.); 18283148194@163.com (C.L.); 17630848120@163.com (X.N.); xjaucy@163.com (Y.C.)

**Keywords:** photoperiod, yellow-feathered broilers, growth performance, serum biochemistry, skeletal development, meat quality

## Abstract

This experiment aimed to investigate the effects of different lighting cycles on the growth performance, serum biochemical indices, slaughter performance, skeletal development, and meat quality of yellow-feathered broiler chickens. A total of 256 10-day-old Liangfenghua yellow-feathered male broilers were randomly assigned to four groups, each with 8 replicates, and each replicate contained 8 chickens. The four lighting cycles used were 18L:6D, 20L:4D, 22L:2D, and 23L:1D (hours of light:dark), and the chickens were fed until 63 days of age. The results indicate that with increasing light exposure duration, the average daily gain and live weight of yellow-feather broilers from 10 to 21 days of age (*p* = 0.07), the average daily feed intake from 42 to 63 days of age (*p* < 0.01), as well as the average daily feed intake, daily gain, and feed-to-gain ratio from 1 to 63 days of age (*p* < 0.05), all showed linear changes. The live weight, daily gain, and feed-to-gain ratio from 42 to 63 days of age exhibited both linear and quadratic variations (*p* < 0.01). The 22L:2D group achieved the highest live weight and daily gain at 63 days, with the lowest feed-to-gain ratio. Serum glucose levels displayed quadratic variation, while phosphorus levels showed both linear and quadratic changes; the 22L:2D group had the lowest serum glucose and the highest phosphorus levels. The semi-eviscerated rate and the bursa of Fabricius index varied linearly, whereas the leg muscle rate showed quadratic variation. The 20L:4D group had the highest total eviscerated rate, breast muscle rate, and leg muscle rate (*p* < 0.05), with the lowest Fabricius and thymus indices. The length, width, and volume of the femur and tibia, as well as the tibia bone volume and tissue volume, all changed linearly (*p* < 0.05). The tibia bone volume fraction exhibited both linear and quadratic changes, with the 18L:6D group showing the highest femur length, width, tibia bone volume, tissue volume, and bone volume fraction. Light exposure significantly affected meat pH, colour, shear force, drip loss, and cooking loss. The crude protein content in the breast muscle showed quadratic variation, while ash and cysteine contents displayed both linear and quadratic changes. The 22L:2D group had the highest crude protein content, pH at 24 h, shear force, and drip loss, but the lowest amino acid and ash contents (*p* < 0.05). Additionally, there were significant differences in the flavour of the breast muscle under different light cycles. In summary, the photoperiod significantly affects the production performance, skeletal development, meat quality, and flavor of yellow-feathered broilers. The 22L:2D photoperiod yielded the best outcomes in promoting growth, improving feed efficiency, and enhancing meat quality; however, it may adversely affect skeletal development and structural integrity, and caution should be exercised for long-term rearing.

## Simple Summary

Light plays an important role in chicken farming. This study looked at how different daily light periods affect the growth, health, and meat quality of yellow-feathered broilers. Chickens were given four different light–dark cycles, from 18 to 23 h of light per day, and raised until 63 days old. The results showed that a 22 h light and 2 h dark schedule (22L:2D) gave the best growth, highest body weight, and best feed use. However, too much light harmed leg bone development. The 22L:2D cycle also increased protein in the breast meat and changed meat quality, including color, tenderness, and water holding. Meat flavor also differed among the light treatments. Overall, the 22L:2D schedule is recommended for raising yellow-feathered broilers over a short period, because it improves growth, feed efficiency, and meat quality. These findings can help farmers choose better lighting to improve production.

## 1. Introduction

Chicken meat is rich in protein, low in fat, and cost-effective, establishing it as a primary source of animal protein for human consumption [1]. As global demand for chicken increases, broiler farming has experienced rapid growth in recent decades [2]. According to the United Nations Food and Agriculture Organization (FAO), global chicken production reached 128 million metric tons in 2024, making it the leading meat product worldwide [3]. In 2024, China produced 14.842 billion broilers, of which fast-growing white-feathered broilers accounted for 11.508 billion birds (77.54%) and slow-growing yellow-feathered broilers accounted for 3.334 billion birds (22.46%) [4]. Yellow-feathered broilers are Chinese indigenous breeds; compared with fast-growing broilers, they exhibit slower growth rates and longer finishing periods. These growth characteristic differences enable yellow-feathered broilers to deposit more flavor compounds and achieve higher protein content [5]. Lighting constitutes a significant environmental factor in intensive broiler production, affecting circadian rhythms, feeding regulation, nutrient metabolism, physiological functions, immune responses, and behavioral responses in chickens [6,7]. Moreover, the sensitivity of poultry to light varies according to breed, age, and genotype [8]. Consequently, lighting cycles in intensive broiler systems have garnered international attention concerning animal health and welfare [9,10].

The light exposure cycle is instrumental not only in enhancing the health and welfare of broiler chickens but also exerts a significant influence on production performance, product quality, and economic advantages [11]. Rapid growth, high feed efficiency, and meat quality serve as key indicators for assessing the production efficiency and economic outcomes within the broiler industry [12]. Conventional broiler production often employs methods such as increasing the light duration to augment feed intake and promote rapid growth [6]; however, this strategy does not invariably improve feed efficiency [13] and may precipitate a variety of health issues and welfare concerns [14]. Furthermore, accelerated growth rates in broilers are associated with an increased incidence of metabolic diseases and skeletal deformities, which can compromise meat quality and elevate mortality rates [7]. Research demonstrated that prolonged light durations could bolster growth rate and overall production performance [15] as well as enhance feed efficiency [10]. Nonetheless, some reports suggested that while extended lighting accelerated growth, it markedly diminished feed efficiency [16]. Additionally, prolonged light exposure influenced the deposition of proteins and lipids within the muscles, potentially adversely affecting meat quality. It has been observed to elevate oxidative stress levels in the breast muscle [17], damage skeletal muscle architecture, induce atrophy [18], increase the prevalence of white striping [19], and provoke abnormalities and inflammation in the breast tissue [16], thereby impairing oxidative stability through alterations in muscle metabolites [20]. Moreover, the skeletal issues resulting from rapid growth in broilers have become increasingly prominent [21]. Bone development in broilers is a complex physiological process governed by multiple factors; besides genetic and nutritional influences, light exposure, as a vital environmental factor, can profoundly affect bone formation and remodelling by modulating endocrine rhythms and metabolic pathways [14]. Research indicated that continuous light exposure could impair leg bone development and diminish their strength in subsequent stages [22], leading to trabecular bone de-generation, inflammation, early osteoporosis-like changes in bone microstructure, and developmental abnormalities in tibial cartilage [23]. These effects may give rise to skeletal deformities, osteoporosis, and mineral metabolism disorders. Consequently, optimizing the light duration to enhance broiler production performance and health has garnered considerable interest. Based on the above research background, we hypothesize that extending the light period may promote growth performance in yellow-feathered broilers by increasing feed intake, but an excessively long light duration could adversely affect skeletal health; simultaneously, a prolonged light period may alter the deposition pattern of muscle proteins and lipids, thereby affecting meat quality. Although some studies have examined the effects of lighting regimes on broiler performance and health, the influence of the light exposure cycle—an essential component of lighting systems—on the performance, skeletal health, and meat quality of yellow-feathered broilers remains insufficiently explored and lacks a comprehensive systematic analysis.

Therefore, using yellow-feathered broilers as experimental animals and simulating the modern intensive cage rearing environment for broilers, this study investigated the effects of different photoperiods of LED white light on growth performance, serum biochemical parameters, slaughter performance, skeletal health, and meat quality, with the aim of evaluating the regulatory effects of photoperiod on economic traits and product quality. This research provides a scientific basis and technical support for optimizing light management regimes in production practice, increasing broiler production efficiency, improving meat quality, and enhancing economic returns.

## 2. Materials and Methods

### 2.1. Ethical Statement

All animal experiments were conducted according to the relevant national guide lines and were approved by the Animal Welfare Care and Use Committee of Xinjiang Agricultural University (Xinjiang, China) (Animal protocol number: 2024005). Sampling operations were strictly conducted in accordance with the relevant provisions of the “Guiding Opinions on the Humane Treatment of Experimental Animals” issued by the Ministry of Science and Technology of the People’s Republic of China (Guo Ke Fa Cai Zi [2006]. No. 398).

### 2.2. Experimental Design and Feeding Management

A total of 300 one-day-old Liangfenghua yellow-feathered broiler cockerels were selected. After a brooding period of 10 days, 256 birds were chosen based on similar body weight and randomly allocated into four groups: 18L:6D, 20L:4D, 22L:2D, and 23L:1D. Each group consisted of 8 replicates, with 8 birds per replicate. All broilers were reared in single-layer stainless steel cages (length × width × height = 120 cm × 70 cm × 55 cm) at a stocking density of 29.5 kg/m^2^. Each cage was equipped with two nipple drinkers and an 80 cm trough installed on one side, and LED lighting was provided at an intensity of 15 lux. Birds were fed the same diets in three phases until 63 days of age. The diets were formulated according to the Chinese agricultural standard NY/T 3645-2020 [24] “Nutrient requirements of yellow feathered broilers”, using standard commercial feeds (510H, 511H, and 512H compound feeds for yellow-feathered large broilers, Xinjiang Taikun Group Co., Ltd., Xinjiang, China), and the ingredient composition and nutrient levels are presented in Table 1. All broilers had ad libitum access to feed and water. Routine management, including vaccination, was performed in accordance with NY/T 1871-2010 [25] “Technical code of practice for yellow-feathered broiler feeding management”.

### 2.3. Determination Indicators and Methods

#### 2.3.1. Growth Performance

At the start of the trial, the fasting weight of each chicken was recorded. At 21, 42, and 63 days of age, the fasting weight and feed consumption of the experimental chickens were recorded again. Using repetitions as experimental units, the average daily feed intake, average daily weight gain, and feed-to-gain ratio were calculated for each group at each stage.

#### 2.3.2. Serum Biochemistry

On day 63 of age, the experimental chickens were subjected to a 12 h fast and weighed. Then, one chicken with a body weight close to the average was selected from each replicate within each group. Blood samples were drawn from the wing vein to facilitate serum separation. An automatic blood biochemical analyzer (TBA-FX8, Canon Medical Systems Corporation, Tochigi, Otawara-shi, Japan) was employed to measure serum levels of total protein, albumin, globulin, blood urea nitrogen, alanine aminotransferase, aspartate aminotransferase, alkaline phosphatase, triglycerides, total cholesterol, high-density lipoprotein cholesterol, low-density lipoprotein cholesterol, glucose, calcium, and phosphorus. Additionally, the albumin-to-globulin ratio was calculated.

#### 2.3.3. Slaughter Performance

After fasting and weighing the experimental chickens at 63 days of age were anesthetized with sodium pentobarbital and exsanguinated via the jugular vein. Slaughter procedures were performed according to the methods outlined in the “Nomenclature, terminology, and measurement statistics of poultry growth performance” [26]. Live weight, carcass weight, semi-eviscerated weight, eviscerated weight, breast muscle weight, thigh muscle weight, and abdominal fat weight were measured. For semi-eviscerated weight, the trachea, esophagus, crop, intestines, spleen, and reproductive organs were removed, while the heart, liver (without gall bladder), kidneys, lungs, proventriculus, gizzard (emptied and without cuticle), and abdominal fat (including leaf fat and fat around the gizzard) were retained. Eviscerated weight was obtained by further removing the heart, liver, proventriculus, gizzard, and abdominal fat from the semi-eviscerated carcass, leaving only the lungs and kidneys. The dressing percentage, eviscerated yield percentage, semi-eviscerated yield percentage, breast muscle percentage, thigh muscle percentage, and abdominal fat percentage were then calculated.

#### 2.3.4. Organ Index

After slaughtering the experimental chickens’ heart, liver, spleen, proventriculus, gizzard, kidneys, testis, thymus, pancreas, and bursa of Fabricius were carefully separated. Surrounding fat and connective tissue were removed, and the organs were quickly blotted dry with filter paper to remove surface moisture. The fresh weight was then measured promptly. The organ index was calculated as the ratio of the organ’s fresh weight (g) to the pre-slaughter live weight (kg), and expressed in g/kg.

#### 2.3.5. Bone Development

##### Determination of Femur and Tibia Morphology and Quality

Following the slaughter of the experimental chickens, the left femur and tibia were carefully excised, and the adhering muscles, soft tissues, and connective tissues were meticulously removed. The weight, length, and width were measured using an analytical balance (AL204, Mettler-Toledo, Shanghai, China) and a digital vernier caliper (JZYQ-001, Ningbo Mingyan Tools Company Limited, Ningbo, Zhejiang, China). Subsequently, the bones were dried in an oven at 105 °C until a constant weight was attained to determine their dry matter content. They were then extracted with petroleum ether in a Soxhlet extractor for 72 h. After defatting, the bones were dried and incinerated in a muffle furnace at 550 °C. The resulting ash was mixed with 20 mL of a hydrochloric acid-water solution (1:3, *v*/*v*), and 0.15 mL of 15 mol/L nitric acid was added to prevent the formation of insoluble calcium phosphate. The mixture was heated on a hot plate to a gentle boil until it became clear. After cooling, it was diluted with distilled water to a final volume of 100 mL and thoroughly homogenized. For phosphorus determination, the spectrophotometric method with ammonium molybdate vanadate (GB/T 6437-2018) [27] was followed, and the reaction system was adapted for microplate analysis. After adding a color reagent, the absorbance was measured at 400 nm using a microplate reader (INFINITE200, Tecan Austria GmbH, Männedorf, Switzerland). Standard solutions for total phosphorus were prepared with a phosphate standard solution, and a calibration curve was generated using the same color development procedure. The total phosphorus content in the samples was determined based on their absorbance readings. For calcium estimation, the o-cresolphthalein complexone (OCPC) colorimetric method was employed: 8-hydroxyquinoline and an alkaline buffer were added, followed by the OCPC chromogenic reagent. After mixing, the reaction proceeded at room temperature for 10 min. The solution was transferred to a microplate, and the absorbance was measured at 570 nm using the microplate reader. Standard calcium solutions were prepared similarly, and a calibration curve was constructed to quantify calcium levels based on the sample absorbance.

##### Histological Measurement of the Tibia

Subsequently, following the slaughter of the experimental chickens, the right tibia was meticulously excised, with muscles, soft tissues, and connective tissues carefully removed. Micro-computed tomography (Micro-CT) analysis was conducted utilizing the NMC-200 scanner (Pingsheng Medical Technology, Kunshan, Jiangsu, China). The scanning parameters were configured as follows: a tube voltage of 80 kV and a current of 0.06 mA; source-to-detector distance set at 410 mm; source-to-object distance at 90 mm; a frame rate of 20 frames per second; a total of 4000 frames; a transverse field of view (FOV) of 50 mm; an axial FOV of 16 mm; a voxel size of 0.05 × 0.05 × 0.05 mm; a resolution of 35 µm; dimensions of 1000 × 1000 × 3427; a CT threshold of 350; reconstruction type FDK; a radial FOV of 100 mm; a maximum axial scanning range of 250 mm; a scan speed of 4 s per bed; and a spatial resolution of less than 1 µm at 10% MTF, with a minimum reconstructed pixel size of 2 µm. Data acquisition was performed using Cruiser V2.0.14.4 software, followed by three-dimensional reconstruction of the original images with Recon V2.0.14.4 software. The targeted region of interest (ROI) was analyzed using Avatar V2.0.14.4 data analysis software. All samples were extracted from the identical region to ensure consistency in analysis and to accurately determine the required parameters, which were subsequently exported.

#### 2.3.6. Meat Quality

##### Determination of Nutritional Components

The ipsilateral breast muscle post-slaughter was sampled for the determination of moisture, crude protein, crude fat, crude ash, and amino acid content. Moisture content was measured using the direct drying method according to GB/T 9695.15-2008 [28], titled “Method for determination of moisture content in meat and meat products.” Crude protein content was determined using a fully automatic Kjeldahl nitrogen analyzer (FOSS Kjeltec 8400, Hillerød, Denmark), in accordance with GB/T 5009.5-2025 [29]. Crude fat content was measured employing the Soxhlet extraction method in accordance with GB/T 5009.6-2003 [30]. Crude ash content was ascertained following the procedures outlined in GB/T 9695.15-2008. Amino acid composition was analyzed using an amino acid analyzer (Hitachi 8900, Tokyo, Japan), aligned with the standards specified in GB/T 18654.11-2008 [31].

##### Determination of Quality Characteristics

Referring to “Determination of livestock and poultry meat quality” (NY/T 1333-2007) [32] and “Recommendations for technical operating procedures for meat quality evaluation of yellow-feathered broiler chickens”, the ipsilateral breast muscle was selected for the measurement of meat color and pH at 45 min and 24 h post-mortem, as well as shear force, cooking loss, and drip loss. Meat color parameters, including lightness (L*), redness (a*), and yellowness (b*), were measured using a portable colorimeter (CR-10, Konica Minolta, Tokyo, Japan). The pH values at 45 min and 24 h post-mortem were measured using a portable pH meter (HI981036, HANNA Instruments, Năsăud, Romania). Shear force was determined using a tenderness tester (C-LM3B, College of Engineering, Northeast Agricultural University, Beijing, China). For cooking loss, a 2.0 cm × 3.5 cm × 5.0 cm meat sample was weighed initially (Y1), then heated in a water bath at 80 °C for 10 min, blotted dry, and weighed again (Y2). Cooking loss (%) was calculated as follows: example 1. For drip loss, a similarly sized meat sample was weighed initially (Y3), stored at 4 °C for 24 h, and then weighed again (Y4). Drip loss (%) was calculated as follows: example 2.

cooking loss (%) = [(Y1 − Y2)/Y1] × 100(1)

drip loss (%) = [(Y3 − Y4)/Y3] × 100.(2)

##### Determination of Flavor Characteristics: Electronic Nose Method

An accurate amount of 5 g of the ipsilateral breast muscle sample was weighed and homogenized using a tissue grinder. The homogenate was then placed into a 40 mL colorless, odorless, transparent headspace sampling bottle and sealed securely. The sample was allowed to equilibrate at 25 °C for 2 h. After volatile compounds had reached equilibrium, detection was performed using the cNose-18 electronic nose system. During the detection process, consistent temperature and humidity within the testing environment were maintained. Each sample was measured in triplicate. The carrier gas was clean air, with a gas flow rate of 1.0 L/min. The cleaning flow rate was 6 L/min. The detection duration was 40 s, the cleaning duration was 60 s, and the detection temperature was maintained at 25 °C. The sensor types and their corresponding representative sensitive substances are detailed in Table 2.

##### Determination of Taste Characteristics: Electronic Tongue Method

An accurate amount of 5 g of the ipsilateral breast muscle sample was weighed, cut into small pieces, and placed into a 100 mL beaker. Then, 50 mL of deionized water was added, and the beaker was sealed with sealing film. The mixture was sonicated for 30 min, followed by magnetic stirring for an additional 30 min. Thereafter, the mixture was filtered through qualitative filter paper, and no less than 20 mL of the filtrate was collected. The sample was then measured using an electronic tongue (cTongue, Shanghai Bosin Industrial Development Company Limited, Shanghai, China). Before and after measurement, the sensor was cleansed with deionized water. The gain coefficients for channels S1, S2, and S3 were set at ×100. The measurement duration was 120 s. Each sample was analyzed in triplicate, and the maximum electrical signal was maintained within the range of ±0.1 to 2.5.

### 2.4. Data Processing

Data were organized using Excel 2021. One-way analysis of variance (ANOVA) was conducted utilizing SPSS 20.0 software, followed by Duncan’s multiple range test. The overall *p* value was derived from the one-way ANOVA. Linear and quadratic *p* values were obtained by partitioning the treatment sum of squares into independent trend components using orthogonal polynomial contrasts and performing F tests against the error mean square. Presenting all three *p* values together allows for a complete description of the dose–response curve shape and avoids erroneous inferences drawn from the overall difference alone. Principal component analysis (PCA) and radar charts were generated using Origin 64 (OriginLab Corporation, Northampton, MA, USA). Pearson correlation analysis and Mantel tests were performed using R software (version 4.2.0) with the ggcor package (version 0.9.8.1). Pearson correlation coefficients were calculated using default parameters. Results are presented as mean and standard error of the mean (SEM). *p* < 0.05 was deemed indicative of a statistically significant difference, whereas *p* < 0.01 denoted a highly significant difference.

## 3. Results

### 3.1. Impact of Various Light Cycles on the Growth Performance of Yellow-Feathered Broilers

As illustrated in Table 3, between 10 and 21 days of age, the average daily gain and live weight of the yellow-feathered broilers increased linearly with the extension of light exposure duration (*p* < 0.05). From 21 to 42 days of age, the different light cycles did not produce significant effects on average daily feed intake, average daily gain, feed-to-gain ratio, or live weight (*p* > 0.05). During the period from 42 to 63 days of age, live weight, average daily gain, and the feed-to-gain ratio exhibited linear and quadratic variations, whereas the average daily feed intake demonstrated a linear change (*p* < 0.05). Specifically, with increased light exposure, there was a tendency for live weight, average daily feed intake, and average daily gain to increase, while the feed-to-gain ratio decreased. The live weight at 63 days in the 22L:2D group was markedly higher than in the 18L:6D and 20L:4D groups (*p* < 0.01), but not significantly different from the 23L:1D group (*p* > 0.05). The average daily feed intake in the 23L:1D group was highly significantly greater than in the 18L:6D and 22L:2D groups (*p* < 0.01), but not significantly different from the 20L:4D group (*p* > 0.05). The average daily gain in the 22L:2D group was highly significantly higher than in the 18L:6D and 20L:4D groups (*p* < 0.01), but not significantly different from the 23L:1D group (*p* > 0.05). The feed-to-gain ratio in the 22L:2D group was markedly lower than in the 18L:6D and 20L:4D groups (*p* < 0.01), but not significantly different from the 23L:1D group (*p* > 0.05). From 1 to 63 days of age, as light exposure increased, the average daily feed intake, average daily gain, and feed-to-gain ratio of the yellow-feathered broilers demonstrated linear changes (*p* < 0.05), with the average daily gain and feed-to-gain ratio also exhibiting quadratic trends (*p* < 0.01). The highest average daily feed intake recorded was 111.68 g per day in the 23L:1D group, the highest average daily gain was 48.58 g per day in the 22L:2D group, and the lowest feed-to-gain ratio was 2.21. Mortality incidence was low across all treatments and was not statistically evaluated due to the low event rate.

### 3.2. The Effect of Different Light Cycles on Serum Biochemical Indices in Yellow-Feathered Broiler Chickens

As demonstrated in Table 4, with increasing light duration, the serum glucose concentration in yellow-feathered broiler chickens “exhibited a quadratic change, initially decreasing and then increasing (*p* < 0.05). The serum glucose level in the 22L:2D group was markedly lower than that in the 18L:6D and 23L:1D groups, but did not differ significantly from the 20L:4D group (*p* > 0.05). Serum phosphorus content exhibited both linear and quadratic variations, initially increasing and then decreasing (*p* < 0.05), with the 23L:1D group displaying highly significantly lower serum phosphorus than the other three groups. Different light cycles exerted some influence on serum total protein, albumin, globulin, the albumin/globulin ratio, urea nitrogen, alanine transaminase, aspartate transaminase, alkaline phosphatase, triglycerides, total cholesterol, high-density lipoprotein, low-density lipoprotein, total bilirubin, and calcium, although these differences did not reach statistical significance (*p* > 0.05).

### 3.3. Effects of Different Light Cycles on the Slaughter Performance of Yellow-Feathered Broilers

As demonstrated in Table 5, with the extension of light duration, the semi-eviscerated yield exhibited a linear decrease (*p* < 0.05), whereas the thigh muscle percentage demonstrated a quadratic variation, initially increasing and subsequently decreasing (*p* < 0.05). The eviscerated yield in the 20L:4D group was significantly higher than that in the 22L:2D group (*p* < 0.05), yet showed no significant difference from the 18L:6D and 23L:1D groups (*p* > 0.05). The breast muscle percentage was markedly higher in comparison to the 18L:6D and 22L:2D groups (*p* < 0.05), but did not differ significantly from the 23L:1D group (*p* > 0.05). No significant difference in thigh muscle percentage was observed between the 20L:4D and 22L:2D groups (*p* > 0.05); however, both were highly significantly higher than those in the 18L:6D and 23L:1D groups (*p* < 0.01). Different light cycles did not exert a significant influence on the dressing percentage or abdominal fat percentage of yellow-feathered broilers (*p* > 0.05).

### 3.4. The Effect of Varying Light Cycles on the Organ Indices of Yellow-Feathered Broiler Chickens

As demonstrated in Table 6, with increasing light duration, the bursa of Fabricius index of yellow-feathered broiler chickens exhibited a linear increase (*p* < 0.01). Among the groups, the bursa of Fabricius index in the 22L:2D group was highly significantly higher than that in the 18L:6D and 20L:4D groups, but did not differ significantly from the 23L:1D group (*p* > 0.05). No significant difference in thymus index was observed among the 18L:6D, 22L:2D, and 23L:1D groups; however, the 22L:2D group was significantly higher than the 20L:4D group (*p* < 0.05). Different light cycles had no significant effects on the heart index, liver index, spleen index, gizzard index, proventriculus index, kidney index, pancreas index, or testis index of yellow-feathered broilers (*p* > 0.05).

### 3.5. The Impact of Various Light Cycles on Skeletal Parameters in Yellow-Feathered Broiler Chickens

As illustrated in Table 7, with increasing light duration, the length and width of the femur and tibia, as well as the ash content of the tibia, all exhibited a linear decrease (*p* < 0.05). No statistically significant differences were observed in femur length and tibia width among the three groups, 18L:6D, 22L:2D, and 23L:1D, though these measures were significantly higher than those in the 22L:2D group. The tibia length in the 18L:6D group was significantly higher than in the 23L:1D group (*p* < 0.05); however, no significant differences were noted when compared to the 20L:4D and 22L:2D groups (*p* > 0.05). The ash content of the tibia in the 22L:2D group was highly significantly higher than in the 20L:4D and 23L:1D groups (*p* < 0.01), while it did not differ significantly from the 18L:6D group (*p* > 0.05). Different light cycles did not exert a significant influence on the weights of the femur and tibia, nor on the calcium and phosphorus content of the tibia (*p* > 0.05). As illustrated in Table 7, with increasing light duration, the length and width of the femur and tibia, as well as the ash content of the tibia, all exhibited a linear decrease (*p* < 0.05). No statistically significant differences were observed in femur length and tibia width among the 18L:6D, 22L:2D, and 23L:1D groups; however, the 18L:6D and 20L:4D groups were significantly higher than the 22L:2D group (*p* < 0.05). The tibia length in the 18L:6D group was significantly higher than in the 23L:1D group (*p* < 0.05); however, no significant differences were noted when compared to the 20L:4D and 22L:2D groups (*p* > 0.05). The ash content of the tibia in the 22L:2D group was highly significantly higher than in the 20L:4D and 23L:1D groups (*p* < 0.01), while it did not differ significantly from the 18L:6D group (*p* > 0.05). Different light cycles did not exert a significant influence on the weights of the femur and tibia, nor on the calcium and phosphorus content of the tibia (*p* > 0.05).

### 3.6. The Impact of Various Light Cycles on Tibia Parameters in Yellow-Feathered Broilers

**Figure 1:**
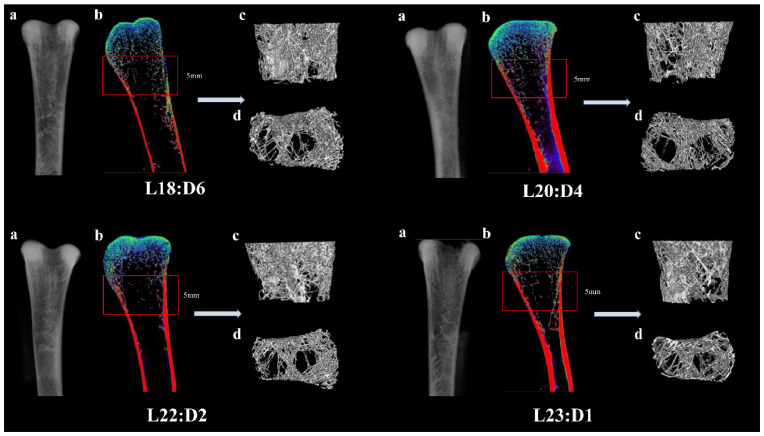
X-ray micro-computed tomography of the tibia structure in 63-day-old yellow-feathered broiler chickens. Figures (**a**–**d**) depict the three-dimensional surface view of the trabeculae, the coloured two-dimensional microstructure, the horizontal view of the trabecular microstructure in the selected region, and the bird’s-eye view of the trabecular microstructure within the selected region, respectively. 18L:6D: 18 h of light and 6 h of dark; 20L:4D: 20 h of light and 4 h of dark; 22L:2D: 22 h of light and 2 h of dark; 23L:1D: 23 h of light and 1 h of dark.

As evidenced in Table 8 and Figure 1, an increase in light exposure duration correlated with a linear decrease in bone volume and tissue volume of the tibia (*p* < 0.05), while bone volume fraction exhibited both linear and quadratic variations (*p* < 0.05). Bone volume in the 18L:6D group was significantly greater than that observed in the 20L:4D, 22L:2D, and 23L:1D groups (*p* < 0.05), whereas no significant differences were identified among the 20L:4D, 22L:2D, and 23L:1D groups (*p* > 0.05). Tissue volume did not differ significantly among the 18L:6D, 20L:4D, and 22L:2D groups (*p* > 0.05); however, the 18L:6D and 22L:2D groups were significantly higher than the 23L:1D group (*p* < 0.05). The highest bone volume fraction was recorded in the 18L:6D group; this did not differ significantly from the 20L:4D and 23L:1D groups (*p* > 0.05). Nonetheless, all three groups demonstrated significantly higher bone volume fractions than the 22L:2D group (*p* < 0.05). The analysis revealed no significant effects of different light cycles on trabecular number, bone mineral density, or bone mineral content (*p* > 0.05).

### 3.7. The Impact of Various Light Cycles on the Quality Attributes of Yellow-Feathered Broiler Breast Muscle

As delineated in Table 9, an increase in light exposure duration resulted in a quadratic variation in pH at 24 h post-mortem and shear force of the broiler breast muscle. Concurrently, the b* value at 45 min post-mortem demonstrated a linear variation, while the a* value at 45 min post-mortem, L* at 24 h post-mortem, and drip loss exhibited both linear and quadratic variations (*p* < 0.05). The pH at 45 min post-mortem for the 20L:4D and 23L:1D groups was significantly higher than that of the 18L:6D group (*p* < 0.05); however, no significant difference was observed when compared with the 22L:2D group (*p* > 0.05). The pH at 24 h post-mortem for the 18L:6D and 22L:2D groups was highly significantly higher than that of the 20L:4D and 23L:1D groups (*p* < 0.01), with the 23L:1D group being highly significantly higher than the 20L:4D group (*p* < 0.01). The a* value at 45 min post-mortem in the 18L:6D group was highly significantly higher than that in the other three groups (*p* < 0.01), with no significant differences observed among the other three groups (*p* > 0.05). The b* value at 45 min post-mortem was highly significantly higher in the 18L:6D group compared to the 22L:2D and 23L:1D groups (*p* < 0.01), but not significantly different from the 20L:4D group (*p* > 0.05). The L* value at 24 h post-mortem in the 23L:1D group was significantly higher than that in the 20L:4D group (*p* < 0.05), but showed no significant differences relative to the 18L:6D and 22L:2D groups (*p* > 0.05). The shear force in the 22L:2D group was highly significantly higher than that in the other three groups (*p* < 0.01). Conversely, the 18L:6D and 20L:4D groups exhibited highly significantly higher shear force than the 23L:1D group (*p* < 0.01), with no significant difference between the 18L:6D and 20L:4D groups (*p* > 0.05). No significant differences in drip loss were observed among the 18L:6D, 20L:4D, and 22L:2D groups (*p* > 0.05); however, all three were highly significantly higher than the 23L:1D group (*p* < 0.01). Similarly, no significant differences in cooking loss were observed among the 18L:6D, 20L:4D, and 22L:2D groups (*p* > 0.05); however, both the 18L:6D and 22L:2D groups were highly significantly higher than the 23L:1D group (*p* < 0.01). Different light cycles did not significantly influence the L* at 45 min post-mortem, a* at 24 h post-mortem, or b* at 24 h post-mortem of the broiler breast muscle (*p* > 0.05).

### 3.8. The Impact of Various Light Cycles on the Nutritional Components of Yellow-Feathered Broiler Chicken Breast Muscle

As demonstrated in Table 10, an increase in light duration resulted in a quadratic variation in the crude protein content of the breast muscle in yellow-feathered broilers (*p* < 0.01), while the ash content exhibited both linear and quadratic variations (*p* < 0.05). The crude protein level in the breast muscle of the 22L:2D group was highly significantly higher than that observed in the 18L:6D and 23L:1D groups (*p* < 0.01), with no statistically significant difference compared to the 20L:4D group (*p* > 0.05). The ash content in the breast muscle of the 18L:6D and 20L:4D groups was highly significantly higher than that of the 22L:2D group (*p* < 0.01), and there was no significant difference when compared to the 23L:1D group (*p* > 0.05). Different light cycle regimens did not significantly influence the moisture or crude fat contents in the breast muscle of yellow-feathered broilers (*p* > 0.05).

### 3.9. The Influence of Various Light Cycles on Amino Acid Composition in the Pectoral Muscle of Yellow-Feathered Broilers

As illustrated in Table 11, with an extension in light duration, the cysteine content within the pectoral muscle of yellow-feathered broilers demonstrated both linear and quadratic variations (*p* < 0.01). The cysteine level in the 20L:4D group was highly significantly higher than those observed in the 18L:6D, 22L:2D, and 23L:1D groups (*p* < 0.01). As the light duration increased, the overall amino acid concentrations (excluding methionine) in the pectoral muscle exhibited a trend of initially decreasing and subsequently increasing (*p* < 0.01). The contents of aspartic acid, threonine, serine, alanine, valine, isoleucine, leucine, tyrosine, phenylalanine, histidine, lysine, and arginine in the 18L:6D group were highly significantly higher than those in the 22L:2D and 23L:1D groups (*p* < 0.01), but did not differ significantly from the 20L:4D group (*p* > 0.05). Moreover, the contents of glutamic acid, proline, and glycine in the 18L:6D group were highly significantly higher than those in the 20L:4D, 22L:2D, and 23L:1D groups (*p* < 0.01). In the 20L:4D group, the concentrations of threonine, serine, alanine, valine, tyrosine, phenylalanine, lysine, and arginine were highly significantly higher than those in the 22L:2D group (*p* < 0.01), but showed no significant difference when compared with the 23L:1D group (*p* > 0.05). Additionally, the aspartic acid level in the 20L:4D group was highly significantly higher than that in the 22L:2D and 23L:1D groups (*p* < 0.01). Nevertheless, the contents of glutamic acid, proline, glycine, and histidine displayed no significant differences in comparison to the 22L:2D and 23L:1D groups (*p* > 0.05). The 22L:2D group recorded the lowest contents of all amino acids except methionine in the pectoral muscle; however, no significant differences were observed relative to the 23L:1D group (*p* > 0.05). Methionine levels did not significantly vary among the four experimental groups (*p* > 0.05).

### 3.10. Effects of Different Light Cycles on Flavor and Taste of Breast Muscle in Yellow-Feathered Broilers

**Figure 2:**
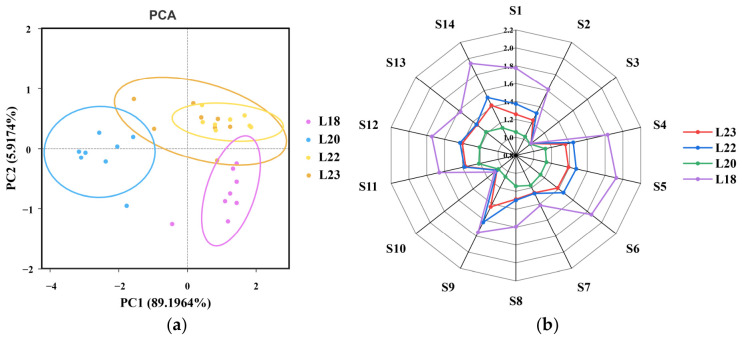
Principal component analysis (PCA) plot (**a**) and radar plot (**b**) of the electronic nose for breast muscle of yellow feathered broilers subjected to different light cycles. 18L:6D: 18 h of light and 6 h of dark; 20L:4D: 20 h of light and 4 h of dark; 22L:2D: 22 h of light and 2 h of dark; 23L:1D: 23 h of light and 1 h of dark.

Figure 2a,b: The results of the principal component analysis regarding the electronic nose assessment of the breast muscle in yellow-feathered broilers subjected to various light cycles are presented in Figure 2a. The two principal components, PCA1 and PCA2, accounted for 89.1964% and 5.9174% of the variance, respectively, with a cumulative contribution of 95.1138%. This suggested that these principal components effectively encapsulated the flavor characteristics of each sample group. The variations between groups were primarily reflected in PC1, which distinctly differentiated the groups, indicating that the samples exhibited significant differences and good repeatability, thus lending credibility to the research findings.

The radar chart illustrating the volatile substances detected by the electronic nose under different light cycles is depicted in Figure 2b. When combined with the response values from each sensor of the electronic nose, the integrated analysis revealed that, in comparison to the 18L: 6D group, the sensor response values of S1, S2, S4, S5, S6, S7, S8, S11, S12, S13, and S14 in the 20L: 4D, 22L: 2D, and 23L: 1D groups were significantly reduced (*p* < 0.05). Conversely, relative to the 20L: 4D group, the sensor response values of S1, S2, S3, S4, S5, S6, S7, S8, S9, S11, S12, S13, and S14 in the 22L: 2D and 23L: 1D groups were significantly elevated (*p* < 0.05).

**Figure 3:**
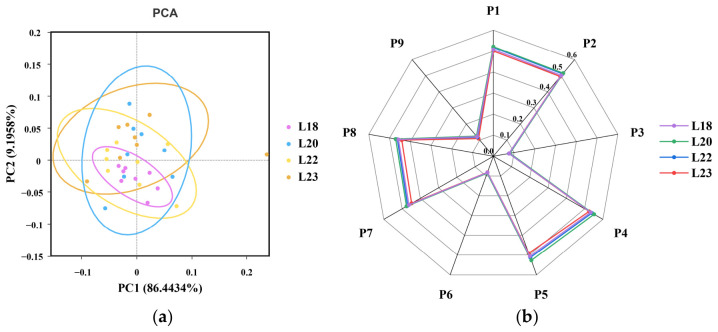
Principal component analysis (**a**) and radar chart (**b**) of the electronic tongue for the breast muscle of yellow-feathered broilers under different lighting cycles. 18L:6D: 18 h of light and 6 h of dark; 20L:4D: 20 h of light and 4 h of dark; 22L:2D: 22 h of light and 2 h of dark; 23L:1D: 23 h of light and 1 h of dark.

Analysis of the electronic tongue PCA plot for breast muscle of yellow-feathered broilers under different light cycles (Figure 3a) showed that the two principal components, PCA1 and PCA2, accounted for 86.4434% and 9.1958% of the total variance, respectively, with a cumulative contribution of 95.6392%. This indicated good repeatability among samples within each group, and the results were reliable. The electronic tongue radar plot for breast muscle of yellow-feathered broilers under different light cycles (Figure 3b) revealed that the response values of all sensors showed no significant differences (*p* > 0.05).

### 3.11. Analysis of Growth Performance and Correlation with Skeletal Parameters

**Figure 4:**
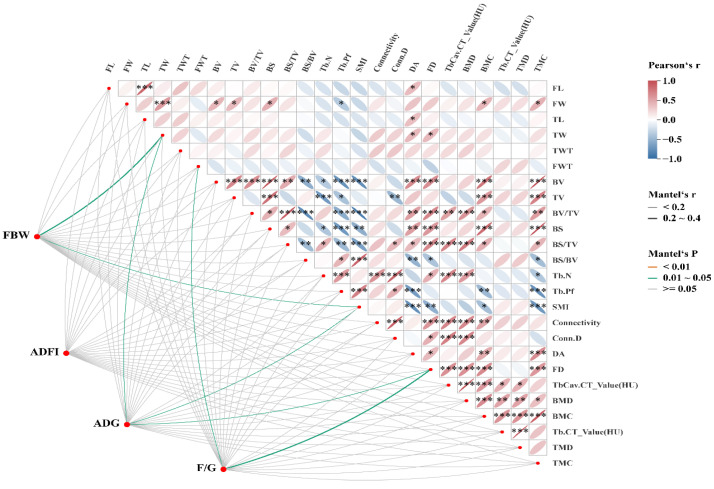
Correlation analysis of growth performance and tibial-related indicators. Green line: Mantel 0.01 ≤ *p* ≤ 0.05; grey line: Mantel *p* ≥ 0.05. * *p* < 0.05; ** *p* < 0.01; *** *p* < 0.001. FBW: Body weight; ADFI: Average daily feed intake; ADG: Average daily gain; F/G: Feed to gain ratio. FL: Femur length; FW: Femur width; TL: Tibia length; TW: Tibia width; TWT: Tibia weight; FWT: Femur weight; BV: Bone volume; TV: Tissue volume; BV/TV: Bone volume fraction; BS: Bone surface area; BS/TV: Bone surface area to tissue volume ratio; BS/BV: Bone surface area to bone volume ratio; Tb.N: Trabecular number; Tb.Pf: Trabecular pattern factor; SMI: Structure model index; Connectivity: Trabecular connectivity; Conn.D: Connectivity density; DA: Degree of anisotropy; FD: Fractal dimension; Tb Cav. CT Value: CT value of the marrow cavity; BMD: Bone mineral density; BMC: Bone mineral content; Tb. CT Value: Trabecular bone CT value; TMD: Tissue mineral density; TMC: Tissue mineral content. 18L:6D: 18 h of light and 6 h of dark; 20L:4D: 20 h of light and 4 h of dark; 22L:2D: 22 h of light and 2 h of dark; 23L:1D: 23 h of light and 1 h of dark.

A correlation analysis was conducted between growth performance and key tibial parameters utilizing Pearson correlation. The findings are illustrated in Figure 4. The feed conversion ratio demonstrated a significant positive correlation with femur weight (*p* = 0.031, *r* = 0.183). Average daily gain showed a positive correlation with tibial width (*p* = 0.016, *r* = 0.184) and the tibial structural pattern index (*p* = 0.026, *r* = 0.189). Final weight was positively correlated with the tibial structural pattern index (*p* = 0.037, *r* = 0.195) and tibial width (*p* = 0.013, *r* = 0.221). Femur width exhibited positive correlations with tibial width (*p* = 0.001, *r* = 0.556), tibial bone volume (*p* = 0.031, *r* = 0.382), tibial tissue volume (*p* = 0.020, *r* = 0.409), and tibial mineral content (*p* = 0.020, *r* = 0.409).

## 4. Discussion

### 4.1. Effects of Different Light Cycles on Growth Performance of Yellow-Feathered Broilers

Light, as one of the most vital environmental parameters in broiler rearing, exerts a significant influence on the growth performance of broilers [33]. Early investigations demonstrated that darkness reduced feed intake in broilers, thereby impeding growth [34], whereas increased light exposure could foster growth development through enhanced feed intake [35]. Consequently, continuous (24L:0D) or nearly continuous (23L:1D) lighting regimens are prevalent in broiler production [36]. Although extending light duration can maximize feed intake and accelerate growth, it does not necessarily enhance feed conversion ratio [13]. The photoperiod drives the central circadian clock via the suprachiasmatic nucleus, regulating the circadian rhythm of melatonin and thereby modulating the physiological window of feeding; however, it disrupts the circadian peaks of insulin and leptin, impairing insulin sensitivity during the melatonin secretion peak, reducing nutrient utilization efficiency, and causing disturbances in energy metabolism [37]. Kim et al. [9] examined the effects of three different continuous light cycles, 8L:16D, 18L:6D, 24L:0D, and intermittent lighting (4L:2D) on the growth metrics of male Ross 308 broilers. Their findings indicated that an increase in continuous light duration correlated with gradual increases in feed intake, weight gain, and final body weight, while the feed conversion ratio declined. Notably, when the light cycle was less than 18L:6D, feed intake showed a significant downward trend. Yu et al. [38] reported that prolonged light duration promoted growth rate, acetylcholine expression, and α4 nAChR mRNA expression, but reduced feed efficiency, inhibited M3 mAChR mRNA expression, and induced glucose metabolism disorders in broilers. Some research suggests that prolonged continuous lighting may inhibit growth, induce physiological stress, and increase mortality in poultry [39]. Jiang et al. [7] analyzed four light cycles, 12L:12D, 16L:8D, 18L:6D, and 20L:4D, and their effects on Ross 308 broilers. Their results revealed that the group subjected to 12L:12D exhibited the highest final body weight and ADG, with a linear decline in these parameters as light duration increased. Ghanima et al. [13] investigated the influence of 3L:1D, 18L:6D, 5L:1D, 20L:4D, 11L:1D, and 22L:2D on Cobb-500 broilers, observing that both continuous and intermittent lighting regimes led to significant increases in final body weight, feed intake, weight gain, and F/G with increased light duration, accompanied by a notable rise in productivity. However, mortality rates also rose progressively. Shynkaruk et al. [10] examined four lighting schedules, 14L:10D, 17L:7D, 20L:4D, and 23L:1D, and their effects on the production performance of Ross 308 broilers raised without antibiotics. Their findings indicated that the 20L:4D group attained the highest ADG and ADFI, with no significant differences in mortality across groups. Conversely, the 23L:1D group experienced the highest mortality due to other causes such as dehydration, twisted necks, and rectal obstruction, suggesting that prolonged continuous lighting compromises health. These studies collectively highlight that darkness is as crucial as light for broiler growth and health, providing essential rest that reduces energy expenditure and stress levels, thereby enhancing feed digestion and promoting overall health [40]. Accordingly, a balanced lighting regimen is vital for optimizing growth performance and ensuring the welfare of broilers. The present study’s findings indicate that the average daily feed intake, average daily gain, and feed-to-gain ratio of yellow-feathered broilers changed linearly with increasing light duration. Lewis et al. [41] investigated the impact of light cycles ranging from 2 to 21 h on the growth performance of Cobb and Ross broiler males, discerning that feed intake and growth rate during the first 21 days were positively correlated with photoperiod. For cycles exceeding 6 h from 22 to 35 days, no effect on feed intake or growth performance was observed, while under photoperiods of 4 to 12 h, mortality increased proportionally with increasing light duration. Schwean-Lardner et al. [42] studied continuous light cycles of 14L:10D, 17L:7D, 20L:4D, and 23L:1D in Ross 308 and Ross 708 broilers, discovering a quadratic relationship between body weight and photoperiod, with the heaviest birds in the 20L:4D group. When the photoperiod was shorter than 18L:6D, feed intake decreased, and mortality increased linearly with increasing light duration. These findings suggest that extending light duration during the early growth phase promotes growth performance; however, as broilers age, the body may develop adaptive adjustments (e.g., hormone synthesis, intestinal development, and nutrient absorption) to different light cycles [43], thus diminishing the impact on growth performance. In the middle and late growth stages, excessively long light exposure may induce stress and consequently affect growth performance, which explains the quadratic relationship observed. A.S. Silva et al. [44] proposed that inappropriate lighting, whether too long or too short, raises stress levels and increases mortality. Mortality is generally associated with broiler strain, growth rate, and management practices. In the present study, mortality occurred infrequently across all photoperiod treatments in yellow-feathered broilers, which differs from findings in fast-growing broiler strains (e.g., Ross, Cobb). This discrepancy may be attributed to differences in the preference of broilers for specific light conditions depending on strain, age, and behavior [45].

### 4.2. The Effects of Various Light Cycles on Serum Biochemical Indices in Yellow-Feathered Broilers

Blood, a vital tissue within animals, plays an integral role in metabolism, nutritional status, and health. Blood indices serve as reflectors of changes in internal metabolic processes and the functional status of specific tissues and organs. Mosleh et al. [46] observed that continuous lighting at 23L:1D, non-intermittent restricted lighting for 7~28 days at 6L:18D (extended to 29~42 days at 23L:1D), and intermittent lighting at 1L:3D did not significantly influence serum TG, cholesterol, lipoproteins, or other lipid profile parameters in Cobb 500 broilers. Ghanima et al. [13] analyzed the effects of lighting schedules (22L:2D, 11L:1D, 20L:4D, 5L:1D, 18L:6D, and 3L:1D) on serum biochemical indices in Cobb-500 chickens, concluding that neither continuous nor intermittent lighting significantly impacted serum TP, ALB, GLB, A/G, total fats, TG, cholesterol, AST, ALT, creatinine, uric acid, or urea levels. Zhang et al. [47] investigated the impact of four lighting regimes (23L:1D, 16L:8D, 8L:4D: 8L:4D, and 8L:16D) on hematological parameters in WOD168 broilers, discovering a significant elevation in white blood cell count in the 23L:1D group compared to the 8L:16D group, and significantly higher T-CHO relative to the 16L:8D group. Levels of BUN, TP, GLB, and GLU exhibited an increasing trend with prolonged light exposure; however, these differences did not attain statistical significance. Kim et al. [9] studied the effects of three continuous lighting schedules (24L:0D, 18L:16D, 8L:16D) and intermittent lighting at 4L:2D in male Ross 308 broilers, noting significant increases in serum T-CHO and AST activity in the 24L:0D and 4L:2D groups compared to the 18L:16D and 8L:16D groups. TG, GLU, TP, and ALT levels did not differ significantly across groups. Saleh et al. [48] examined the effects of continuous 24L lighting and three variants of intermittent lighting on Rose 308 broilers, observing no significant influence on serum TP and ALB, but noting significant differences in T-CHO, TG, HDL-C, LDL-C, and very low-density lipoprotein levels. The group subjected to continuous 24L lighting exhibited the highest serum GLU levels, aligning with the findings of the present study. The serum glucose concentration was highest in the 23L:1D group, which was significantly higher than that in the other groups. This may be because longer light exposure increased feeding frequency, thereby resulting in elevated blood glucose levels.

### 4.3. The Effects of Different Light Cycles on the Slaughter Performance and Organ Indices of Yellow-Feathered Broilers

Slaughter performance is a critical economic trait in broiler production and serves as a key indicator for assessing the efficacy of rearing practices. The slaughter performance of broilers is influenced by various lighting parameters, including photoperiod, light intensity, and light wavelength [49,50]. Otlu et al. [51] examined the effects of four light cycles (23L:1D, 4L:2D, 8L:4D, and 16L:8D) on the slaughter performance of Ross 308 broilers, concluding that the group subjected to a 16L:8D cycle exhibited lower carcass weight, dressed weight, and breast muscle weight. Ghanima et al. [13] demonstrated that, in comparison to intermittent lighting, continuous 22 h lighting substantially increased dressing percentage, breast muscle percentage, and the proportions of liver, intestine, and abdominal fat in Cobb-500 broilers, primarily attributable to increased pre-slaughter body weight, which is positively correlated with dressing percentage. However, neither continuous nor intermittent lighting, nor varying light intervals (22, 20, or 18 h), significantly affected the weights of thigh muscles, shoulder, left rib, gizzard, or spleen. Reducing daily light exposure from 22 h to 18 h can decrease abdominal fat content and enhance carcass quality, possibly due to diminished feed intake during dark periods, resulting in reduced abdominal fat deposition [52]. Kim et al. [9] investigated the effects of three continuous light cycles (24L:0D, 18L:6D, 8L:16D) and one intermittent cycle (4L:2D) on the slaughter performance of male Ross 308 broilers, finding no significant effects on dressing percentage, breast muscle percentage, thigh muscle percentage, or yields of wings, neck, and back. In the present study, with increasing light duration, the semi-eviscerated yield of yellow-feathered broilers showed a linear decrease, while the thigh muscle percentage exhibited a quadratic pattern, initially increasing and then decreasing. This may be because prolonged light exposure promotes activity, which enhances the development of leg muscles. Different light cycles had no significant effects on dressing percentage or abdominal fat percentage in yellow-feathered broilers, likely owing to increased activity levels that elevate energy expenditure and reduce abdominal fat deposition. These results align with those reported by Downs et al. [35] and Fidan et al. [40]. Khonitan et al. [53] compared 24L:0D, 18L:6D, and two variable lighting programs, noting that continuous lighting or the absence of specific lighting schemes often increased the risk of carcass damage, thereby detracting from carcass quality. In addition to photoperiod, light source and light wavelength also affect broiler slaughter performance. Pap et al. [54] observed that LED lighting could enhance dressing percentage, breast muscle percentage, and thigh muscle percentage in Cobb-500 broilers compared to incandescent lighting, although these differences did not reach statistical significance. Perretti et al. [55] compared the effects of white, blue, and green light on slaughter performance of Cobb 500 broilers, finding no significant differences in live weight at 42 days among groups, but recording higher dressing percentages under white and green light than under blue light, with white light producing the highest breast muscle percentage and improving carcass quality. Conversely, Tekin et al. [56] found that daylight, warm white, and blue LED lighting had no significant effects on dressing percentage or the proportions of heart, liver, gizzard, and abdominal fat in Ross 308 broilers. These discrepancies may be attributable to variations in broiler breeds, experimental conditions, and dietary nutrient levels, with the underlying mechanisms warranting further investigation.

Organ indices, to some extent, reflect the growth and development status as well as immune function [57]. The thymus and bursa of Fabricius are both important central immune organs. The thymus plays a critical role in the development and maturation of T lymphocytes; it grows gradually with age and matures during the juvenile period (2–7 weeks of age) [58]. The bursa of Fabricius is a vital organ for the development and maturation of B lymphocytes [59]. Studies have shown that green and blue light significantly enhance cell proliferation, development, and immune function in the thymus and bursa of Fabricius by regulating melatonin receptor expression and antioxidant systems; in contrast, red light weakens immune defense capabilities by activating nuclear receptors and inhibiting membrane receptor pathways. The effect of white light is intermediate between the two or similar to that of red light [60]. In the present study, with increasing photoperiod, the bursa of Fabricius index of yellow-feathered broilers increased linearly, and the thymus index in the 22L:2D group was significantly improved, while no significant changes were observed in other organ indices. This outcome may be attributed to the stress response induced by prolonged light duration, which in turn stimulates changes in the immune system [61].

### 4.4. The Effects of Different Light Cycles on Skeletal Health in Yellow-Feathered Broiler Chickens

Bone quality is an important welfare, health, and economic indicator in poultry production. Impaired bone development can lead to increased mortality, reduced production performance, and consequent economic losses [62]. Light is a key environmental factor modulating skeletal health in broilers, and an appropriate photoperiod can enhance bone strength and mineral deposition by promoting osteogenic activity during the light phase and reducing bone resorption during the dark phase [63]. Studies have shown that metabolic and skeletal diseases, as well as total mortality, increase linearly with increasing light duration. Therefore, the development of bone tissue must be coordinated with the rate of muscle deposition to support the rapidly increasing body weight and thereby reduce the incidence of leg disorders caused by excessively rapid growth [64,65]. In modern broilers, the high-speed growth of muscle tissue compromises skeletal health, predisposing birds to diseases such as tibial dyschondroplasia (TD) [23]. Freitas et al. [21] pointed out that although prolonged light exposure can promote growth rate and mineral deposition by increasing feed intake, it may also increase the risk of tibial deformation, reduce compressive strength, and induce microstructural changes in bone characteristic of early osteoporosis, thus being detrimental to bone health. The length, diameter, mineral deposition, and strength of the femur and tibia directly determine the standing, walking, and feeding ability of broilers [66]. Bone mineral content, bone density, bone area, and ash content are commonly used as key indicators for evaluating bone quality [67]. Jiang et al. [7] studied the effects of different photoperiods on the skeletal health of Ross 308 broilers and found that the leg bone health of birds in the 12L and 16L groups was superior to that in the 18L and 20L groups, with the 12L group exhibiting higher bone density and bone mineral content, longer and wider femurs, and a lower femur length-to-width ratio (H/L). The results of the present study showed that with increasing continuous light duration, both the length and width of the femur and tibia in yellow-feathered broilers tended to decrease. Femur length and tibia width in the 22L:2D group were significantly lower than those in the 18L:6D and 20L:4D groups, and tibia length in the 23L:1D group was significantly lower than that in the other groups. Bone mineral content is positively correlated with ash content; the higher the ash content, the greater the compressive strength and rigidity of the bone, and the lower the risk of leg problems [65]. The present study found that with increasing photoperiod, the tibia ash content of yellow-feathered broilers decreased linearly, whereas no significant effects were observed on tibia calcium or phosphorus content. Bone volume, bone volume fraction, and trabecular number are core parameters for evaluating bone microstructure and skeletal health, determining the mechanical strength and fracture resistance of bone [68]. Prolonged light exposure can reduce the number of trabeculae in the tibia and alter its microstructure [69]. In the present study, the bone volume fraction in the 18L:6D group was significantly higher than that in the 22L:2D group, and the tissue volume in the 23L:1D group was significantly lower than that in the 18L:6D and 22L:2D groups. These results indicate that excessively long light duration adversely affects the growth and development of the femur and tibia in yellow-feathered broilers, and that an 18L:6D photoperiod is more conducive to maintaining macroscopic and microscopic bone structural integrity. The results of this experiment also showed that serum phosphorus content in the 23L:1D group was highly significantly lower than that in the 18L:6D, 20L:4D, and 22L:2D groups, while serum calcium content did not differ significantly among groups. Combined with the bone parameter results, it is speculated that excessively long light duration may interfere with phosphorus metabolism and utilization, thereby affecting the bone mineralization process. This may be because the photoperiod influences the dynamic balance between osteoblasts and osteoclasts by modulating pineal melatonin secretion and hypothalamic-pituitary-growth axis (GH/IGF-1) activity [70]. Moreover, circadian clock genes (e.g., Clock, Bmal1) play a key role in the regulation of bone metabolism; disruption of the photoperiod may disturb the local circadian rhythm in bone tissue, leading to dysregulation of the bone tissue clock and affecting the differentiation of osteoblasts and osteoclasts, thereby influencing bone matrix mineralization and remodeling processes [71,72]. Excessively long light duration may induce a stress response, activate osteoclast activity, enhance bone resorption, and lead to bone loss [73]. As can be seen from the above, osteoclast overactivation may be a key link connecting photoperiod with bone microstructural damage, and its specific mechanism still requires further investigation.

### 4.5. The Effect of Different Light Cycles on Meat Quality of Yellow-Feather Broilers

The meat quality of poultry is influenced by light cycles. Different lighting patterns (such as continuous light, intermittent light, and variable photoperiods) affect the physiological rhythms, endocrine status, and metabolic processes of broilers, ultimately resulting in varied effects across multiple aspects of meat quality [16,17,20,40,54,74]. An appropriate light cycle can provide a stable internal environment for the proliferation and differentiation of muscle satellite cells, thereby promoting muscle fibre growth and development and increasing the protein content in the muscle [75]. Conversely, longer light durations can increase oxidative stress levels in the muscle, leading to elevated malondialdehyde levels, damaging muscle morphology, reducing muscle protein content, and increasing fat deposition [51]. Kim et al. [9] studied the effects of four different lighting schemes, 24L:0D (continuous light), 18L:6D, 8L:16D, and intermittent lighting (4L:2D), on the carcass characteristics of Ross 308 male broilers. They found no significant differences in the moisture, crude protein, and crude fat contents of the breast muscle among the groups, except that the shear force was lowest in the 8L:16D group. The light cycle did not affect the pH, meat colour, cooking loss, or water-holding capacity of the broiler breast muscle. Gündoğar et al. [76] examined the effects of LED light–dark cycle mutations, 30 min transitions, and 1 h transitions on the meat quality of Ross-308 broilers. They found that in groups with transitional lighting between light and dark periods, the thigh muscles had higher proportions of protein and fat, lower water content, and the 1 h transition group exhibited the highest water-holding capacity. This may be due to reduced activity of broilers with light–dark transitions, leading to energy being stored as fat in the thigh muscles, and muscle fibre types adjusting internal metabolic structures, affecting metabolism and water-binding properties. Yu et al. [16] investigated the effects of three light cycles (12L:12D, 18L:6D, and 24L:0D) on the morphology of the breast muscle in Arbor Acres broilers. They observed that with increasing light duration, muscle bundle damage and atrophy occurred, accompanied by increased gaps, nuclear migration, and proliferation of fibroblasts in the interstitial tissue, indicating that prolonged light exposure damages breast muscle morphology and causes inflammatory cell infiltration. The current study shows that four light cycles (18L:6D, 20L:4D, 22L:2D, and 23L:1D) had no significant effect on the water and crude fat content in the breast muscle of yellow-feather broilers. However, as the light duration increased, the levels of aspartic acid, threonine, serine, glutamic acid, proline, glycine, alanine, valine, isoleucine, leucine, tyrosine, phenylalanine, histidine, lysine, and arginine in the breast muscle gradually decreased, indicating that extended light exposure is unfavourable for protein deposition in the meat, consistent with the findings of Tuell et al. [17,20]. Tuell et al. [17,20] studied four light cycles (20L:4D, 18L:6D, 16L:8D, and 12L:12D) and found that moisture, crude protein, and crude fat contents, as well as pH, water-holding capacity, and shear force of the breast muscle, were unaffected by the light cycle. However, muscle colour and oxidative stability were influenced; compared to 12L:12D, muscles in the 20L:4D group appeared lighter and more faded, with higher lipid oxidation and protein denaturation. This suggests that while the initial meat quality is not significantly affected by the light cycle, prolonged light durations may negatively impact oxidative stability by altering muscle metabolic products. We found that the crude protein content in the breast muscle of the 22L:2D group was high while its amino acid content was low. The possible reasons are as follows: the crude protein assay employs the Kjeldahl method, which determines total nitrogen content and then multiplies by a conversion factor; therefore, the crude protein value includes not only true amino acid nitrogen but potentially also non-protein nitrogen such as free amino acids, nucleic acids, biogenic amines, and amides. Prolonged light exposure may have elevated the levels of certain non-protein nitrogenous compounds in muscle, thereby increasing the crude protein value, but this does not equate to an increase in total amino acids [77]. Alternatively, the metabolic distribution of amino acids in the body may also play a role. The 22L:2D group exhibited the fastest growth rate, and the body may need to transport more specific amino acids (e.g., those used for skeletal muscle deposition) to tissues; dynamic changes in the free amino acid pool in blood or muscle could lead to lower measured amino acid contents [78]. The specific mechanisms still require further investigation. The present study found that pH at 45 min post-mortem was lowest in the 18L:6D group and highest at 24 h, indicating that broilers under shorter light cycles stored more glycogen in muscles, leading to faster and stronger post-mortem glycolysis. The rapid pH decline at 45 min may cause muscle fibre contraction and impair protein function, reducing water retention [79]. Conversely, the higher pH at 24 h suggests slower anaerobic glycolysis of stored glycogen, resulting in less water exudation and better meat preservation [80]. pH also influences muscle colour, tenderness, and water-holding capacity [81]. The colour of chicken meat is a key sensory attribute and significantly affects purchasing decisions [46]. Factors affecting meat colour include pigment (myoglobin and haemoglobin) content, slaughter timing, processing and storage conditions, muscle slicing, cutting direction, fat content, water status, and the animal’s breed, sex, and age [82]. The L* value mainly reflects muscle brightness; an increase in L* is associated with denaturation and aggregation of myofibrillar proteins, altering the surface reflectance of the meat [83]. Overall, L* values increased with longer light cycles, possibly due to increased activity in broilers, affecting myofibrillar protein composition. The a* value reflects the oxidation state of myoglobin and haemoglobin in the muscle [84], while the b* value indicates freshness [85]. The 18L:6D group showed the highest a* 45 min and b* 45 min values, indicating better colour and freshness, and thus higher meat quality. The 22L:2D group had the highest shear force, which, along with cooking loss, showed a trend of initially increasing and then decreasing; the 23L:1D group exhibited the lowest drip loss and cooking loss. Tenderness is measured by shear force; lower shear force indicates more tender meat, while higher shear force indicates tougher meat. As light exposure increases, broilers become more active, leading to thicker muscle fibres and higher shear force. Increased glycolysis and lactic acid accumulation in muscle tissue lower pH; when pH reaches the isoelectric point of myoglobin, the distance between thick and thin filaments and sarcomere length decrease, causing water to be expelled from the myofibril gaps and increasing drip loss [82]. Drip loss and cooking loss reflect the water-holding capacity of the muscle; approximately 88~95% of water in muscle is retained between actin and myosin filaments within cells, with the remainder located between myofibrils. Light exposure may influence the growth of skeletal muscle fibres, thereby affecting water retention capacity. Increased muscle water content can improve tenderness, juiciness, firmness, and appearance, enhancing meat quality and economic value [86]. Generally, higher fat content in muscle correlates with greater drip and cooking losses [87]. Liu [88] studied the effects of different light durations on the meat quality of Camellia chickens, showing that longer light exposure increased cooking loss, water loss, and shear force, indicating that extended light durations may cause water loss and reduce tenderness. Additionally, prolonged light exposure can darken or whiten muscle colour, which is unfavourable for maintaining a bright red appearance. Therefore, extended light durations may deteriorate meat quality and affect muscle integrity. Flavor is a key indicator for evaluating meat quality, formed by the synergistic action of volatile organic compounds and non-volatile metabolites [89]. The electronic nose and electronic tongue provide information from the ‘odor’ and ‘taste’ dimensions, respectively, and their combined use enables a comprehensive evaluation of meat flavor. The studies by Surányi et al. [90,91] have confirmed the effectiveness of the electronic tongue in distinguishing taste differences among different beef breeds; based on the complementarity of odor and taste information, it is necessary to combine the electronic nose and electronic tongue in future studies to establish a more comprehensive, multi-dimensional evaluation system for meat flavor quality. Furthermore, this study used electronic nose and electronic tongue analyses to evaluate the effects of different light cycles on the flavour and taste characteristics of yellow-feather broiler breast meat. Results showed significant differences in electronic nose responses under various light regimes, indicating that light cycle influences chicken meat flavour. This phenomenon may be due to two mechanisms: firstly, differing light durations alter broiler activity levels, affecting the composition of muscle proteins and fats, as well as the types and contents of amino acids and fatty acids involved in synthesis; secondly, longer light exposure may promote changes in metabolic synthesis and breakdown, leading to variations in volatile flavour compounds such as alcohols and aldehydes. On the one hand, the metabolism of alanine, aspartic acid, and glutamic acid can regulate flavor metabolism by affecting the production of amino acid-derived compounds, and through participating in Strecker degradation, deamination, and decarboxylation reactions, play an important role in the formation of key flavor compounds [92]. Different light durations alter broiler activity levels, thereby influencing changes in muscle protein and fat content as well as the types and contents of amino acids and fatty acids involved in protein and fat synthesis. On the other hand, the oxidation of linoleic acid and arachidonic acid produces flavor precursors such as aldehydes and ketones, and disturbances in these metabolic pathways lead to the accumulation of oxidation products (e.g., hexanal and nonanal), resulting in flavor differences [93]. Excessive glycerophospholipid metabolism disrupts cell membrane integrity in tissues, and the released fatty acids undergo oxidation, altering meat flavor [94]. A longer photoperiod may promote changes in lipid synthesis and catabolism, causing variations in volatile flavor compounds such as alcohols and aldehydes. These hypotheses require further investigation through detailed material basis studies.

## 5. Conclusions

In conclusion, extending the light duration increased feed intake, daily gain, and feed efficiency of yellow-feathered broilers. For fast-growing yellow-feathered broilers marketed before 70 days of age, the 22L:2D photoperiod achieved the highest final body weight and the lowest feed-to-gain ratio without causing excessive mortality. However, prolonged light exposure negatively affected skeletal health, as evidenced by reduced femoral and tibial dimensions, decreased bone volume fraction, lowered serum phosphorus levels, and compromised tibial microstructural integrity. Lighting cycle also significantly influenced slaughter performance and meat quality: the 20L:4D group showed the highest dressed percentage, breast muscle percentage, and leg muscle percentage, while the 22L:2D group exhibited greater breast muscle crude protein content and water-holding capacity. Optimal bone development was observed under the 18L:6D photoperiod. Taking growth performance, skeletal health, and meat quality together, the 22L:2D photoperiod can be recommended as a short-term fattening strategy prior to market at 70 days of age, whereas for long-term rearing beyond 70 days, the 18L:6D photoperiod is more favorable from the perspectives of skeletal integrity and animal welfare. Furthermore, phased lighting strategies that apply a longer photoperiod during the starter phase to maximize early growth and then transition to a moderate photoperiod to protect skeletal development represent a promising direction for future research to balance production performance and skeletal health.

## Figures and Tables

**Table 1:** Composition and nutrient levels of diets (air-dry basis).

Items	Start (1–21 Days)	Grower (22–42 Days)	Finisher (43–63 Days)
Ingredients (%)			
Corn	59.35	56.17	58.48
Cottonseed meal	7	5	4.8
Soybean meal	12.2	7.8	5
Corn protein meal	5	5	5
Wheat flour	5	10	10
Wheat bran	0	3	3.5
Corn germ meal	6.4	7.7	7.3
CaHPO_4_	1.24	1.02	0.76
Limestone	1.39	1.26	1.17
Cottonseed oil	0.6	1.4	2.4
NaCl	0.25	0.2	0.2
Lysine	0.53	0.53	0.49
Methionine	0.2	0.16	0.12
Threonine	0.13	0.1	0.1
Premix ^1^	0.71	0.66	0.68
Total	100	100	100
Calculated nutrient levels ^2^			
ME (MJ/kg)	12.95	13	13.05
CP	18.62	17.26	17.18
Ca	0.9	0.8	0.7
TP	0.5	0.35	0.3
Lysine	1.2	1	0.9
Methionine	0.6	0.5	0.35
Met + Cys	0.92	0.86	0.74

^1^ The premix provides the following per kg of diets: Mn 78 mg, Zn 72 mg, Cu 8 mg, Se 0.20 mg, I 0.40 mg, vitamin A 12,000 IU, vitamin D3 2500 IU, vitamin E 15 IU, vitamin K3 2.2 mg, vitamin B_1_ 2.2 mg, vitamin B_2_ 5.5 mg, vitamin B12 0.02 mg, nicotinic acid 35 mg, pantothenic acid 12 mg, folic acid 1.2 mg, biotin 0.15 mg, choline chloride 1200 mg. ^2^ Crude protein (CP) were measured values, while the others were calculated values.

**Table 2:** Name of the electronic nose sensor and the corresponding representative sensitive substance.

Sensor	Representative Sensitive Substances
S1	propane, smoke
S2	alcohol, smoke, isobutane, formaldehyde
S3	ozone
S4	hydrogen sulfide
S5	ammonia
S6	toluene, acetone, ethanol, hydrogen
S7	methane, natural gas, biogas
S8	liquefied petroleum gas
S9	toluene, formaldehyde, benzene, alcohol, acetone
S10	hydrogen
S11	liquefied petroleum gas, alkanes
S12	liquefied petroleum gas, methane
S13	methane
S14	combustible gas, smoke

**Table 3:** The effect of different light cycles on the growth performance of yellow-feathered broiler chickens.

Items	Light Cycle				SEM	*p*-Value		
	18L:6D	20L:4D	22L:2D	23L:1D		Overall	Linear	Quadratic
10 to 21 days of age								
BW10 (g)	198.04	206.43	198.82	207.11	1.64	0.081	0.210	0.647
BW21 (g)	532.00	543.11	562.61	563.01	5.07	0.070	0.010	0.937
ADFI (g/d)	59.74	60.23	61.45	62.18	0.61	0.487	0.133	0.746
ADG (g/d)	30.36	30.61	33.07	32.36	0.43	0.060	0.018	0.938
F/G	1.98	1.98	1.87	1.94	0.03	0.607	0.375	0.882
Mortality (%)	0	0	0	1.79				
22 to 42 days of age								
BW42 (g)	1734.84	1740.03	1746.83	1737.01	10.06	0.980	0.845	0.785
ADFI (g/d)	123.98	123.13	125.68	124.75	0.41	0.144	0.145	0.543
ADG (g/d)	57.28	57.00	56.39	55.90	0.42	0.671	0.233	0.779
F/G	2.17	2.17	2.23	2.23	0.02	0.239	0.065	0.065
Mortality (%)	3.57	1.79	3.57	1.79				
43 to 63 days of age								
BW63 (g)	2715.32 ^Cc^	2829.26 ^Bb^	2928.58 ^Aa^	2865.19 ^ABab^	20.12	<0.001	<0.001	0.049
ADFI (g/d)	141.16 ^Cc^	145.68 ^ABab^	141.96 ^BCbc^	148.12 ^Aa^	0.83	0.004	0.019	0.909
ADG (g/d)	46.69 ^Bb^	51.87 ^Bb^	56.27 ^Aa^	53.72 ^ABab^	0.83	<0.001	<0.001	0.025
F/G	3.05 ^Aa^	2.81 ^ABab^	2.53 ^Cc^	2.76 ^BCbc^	0.05	<0.001	<0.001	0.043
Mortality (%)	3.87	1.79	0	0				
10 to 63 days of age								
ADFI (g/d)	108.29 ^b^	109.68 ^ab^	109.70 ^ab^	111.68 ^a^	0.44	0.047	0.012	0.67
ADG (g/d)	44.78 ^Cc^	46.49 ^Bb^	48.58 ^Aa^	47.33 ^ABab^	0.36	<0.001	<0.001	0.084
F/G	2.40 ^Aa^	2.32 ^Aa^	2.21 ^Bb^	2.31 ^ABab^	0.02	0.010	0.012	0.094
Mortality (%)	5.36	3.57	3.57	1.79				

SEM: standard error of the mean. In the same row, values marked with different lowercase superscript letters are significantly different *p* < 0.05; those marked with different uppercase superscript letters are highly significantly different *p* < 0.01; and values without a letter or sharing the same letter are not significantly different *p* > 0.05. BW: Body weight; ADFI: Average daily feed intake; ADG: Average daily gain; F/G: Feed to gain ratio. 18L:6D: 18 h of light and 6 h of dark; 20L:4D: 20 h of light and 4 h of dark; 22L:2D: 22 h of light and 2 h of dark; 23L:1D: 23 h of light and 1 h of dark.

**Table 4:** Effects of different light cycles on serum biochemical indices in yellow-feathered broiler chickens.

Items	Light Cycle				SEM	*p*-Value		
	18L:6D	20L:4D	22L:2D	23L:1D		Overall	Linear	Quadratic
TP (g/L)	35.39	35.74	34.99	35.89	0.66	0.968	0.883	0.627
ALB (g/L)	13.55	13.54	13.05	13.54	0.23	0.852	0.720	0.454
GLB (g/L)	21.84	22.20	21.94	22.35	0.48	0.983	0.953	0.760
A/G	0.63	0.61	0.60	0.61	0.01	0.811	0.906	0.782
BUN (mmol/L)	0.13	0.14	0.17	0.07	0.02	0.273	0.455	0.200
ALT (U/L)	1.86	2.25	2.38	1.75	0.13	0.264	0.966	0.079
AST (U/L)	270.13	263.88	275.50	251.88	4.85	0.362	0.277	0.144
ALP (U/L)	986.25	1258.25	1161.88	1009.75	74.70	0.545	0.353	0.928
TBIL (mmol/L)	15.21	14.78	16.33	15.73	0.48	0.709	0.450	0.793
GLU (mmol/L)	11.16 ^Bb^	9.87 ^BCbc^	8.32 ^Cc^	13.17 ^Aa^	0.45	<0.01	0.450	<0.01
TG (mmol/L)	1.35	1.25	1.32	1.56	0.07	0.475	0.361	0.226
T-CHO (mmol/L)	3.47	3.75	3.68	3.90	0.09	0.443	0.171	0.845
HDL-C (mmol/L)	2.37	2.46	2.31	2.39	0.05	0.653	0.955	0.545
LDL-C (mmol/L)	0.89	0.91	0.96	0.90	0.04	0.937	0.795	0.710
Ca (mmol/L)	2.67	2.77	2.73	2.76	0.03	0.458	0.247	0.476
P (mmol/L)	2.50 ^Aa^	2.48 ^Aa^	2.68 ^Aa^	1.88 ^Bb^	0.07	<0.01	0.014	0.002

SEM: standard error of the mean. In the same row, values marked with different lowercase superscript letters are significantly different *p* < 0.05; those marked with different uppercase superscript letters are highly significantly different *p* < 0.01; and values without a letter or sharing the same letter are not significantly different *p* > 0.05. TP: Total protein; ALB: Albumin; GLB: Globulin; A/G: Albumin to globulin ratio; BUN: Blood urea nitrogen; ALT: Alanine aminotransferase; AST: Aspartate aminotransferase; ALP: Alkaline phosphatase; TBIL: Total bilirubin; GLU: Glucose; TG: Triglycerides; T-CHO: Total cholesterol; HDL-C: High-density lipoprotein cholesterol; LDL-C: Low-density lipoprotein cholesterol; 18L:6D: 18 h of light and 6 h of dark; 20L:4D: 20 h of light and 4 h of dark; 22L:2D: 22 h of light and 2 h of dark; 23L:1D: 23 h of light and 1 h of dark.

**Table 5:** Effects of different lighting cycles on the slaughter performance of yellow-feathered broilers (%).

Items	Light Cycle				SEM	*p*-Value		
	18L:6D	20L:4D	22L:2D	23L:1D		Overall	Linear	Quadratic
Dressing percentage	91.81	91.66	91.09	91.47	0.16	0.402	0.201	0.661
Semi-eviscerated yield	85.19	84.49	83.32	83.06	0.40	0.194	0.033	0.936
Eviscerated yield	67.06 ^ab^	69.84 ^a^	65.50 ^b^	68.61 ^ab^	0.56	0.031	0.894	0.526
Breast muscle percentage	19.41 ^b^	21.96 ^a^	18.98 ^b^	19.96 ^ab^	0.36	0.022	0.270	0.122
Thigh muscle percentage	14.62 ^Bb^	15.73 ^Aa^	15.44 ^Aa^	14.59 ^Bb^	0.15	0.005	0.913	<0.001
Abdominal fat percentage	3.40	3.60	3.19	3.20	0.09	0.334	0.223	0.323

SEM: standard error of the mean. In the same row, values marked with different lowercase superscript letters are significantly different *p* < 0.05; those marked with different uppercase superscript letters are highly significantly different *p* < 0.01; and values without a letter or sharing the same letter are not significantly different *p* > 0.05. 18L:6D: 18 h of light and 6 h of dark; 20L:4D: 20 h of light and 4 h of dark; 22L:2D: 22 h of light and 2 h of dark; 23L:1D: 23 h of light and 1 h of dark.

**Table 6:** Effects of different light cycles on organ indices of yellow-feathered broiler chickens (g/kg).

Items	Light Cycle				SEM	*p*-Value		
	18L:6D	20L:4D	22L:2D	23L:1D		Overall	Linear	Quadratic
Heart index	6.69	6.31	6.37	6.18	0.14	0.617	0.248	0.714
Liver index	23.71	23.23	20.90	22.78	0.39	0.061	0.079	0.396
Spleen index	1.65	1.80	1.97	1.88	0.06	0.276	0.082	0.505
Gizzard index	10.97	9.91	10.89	11.05	0.26	0.562	0.719	0.222
Proventriculus index	3.76	3.92	3.63	4.71	0.20	0.232	0.231	0.323
Kidney index	4.88	4.34	4.43	4.56	0.12	0.378	0.343	0.796
Testis index	2.52	2.71	3.33	2.89	0.16	0.333	0.178	0.178
Thymus index	2.01 ^ab^	1.49 ^b^	2.45 ^a^	1.87 ^ab^	0.11	0.021	0.449	0.446
Pancreas index	1.71	1.89	1.89	1.92	0.05	0.427	0.148	0.490
Bursa of Fabricius index	1.22 ^BCbc^	0.96 ^Cc^	1.92 ^Aa^	1.61 ^ABab^	0.09	<0.001	0.002	0.256

SEM: standard error of the mean. In the same row, values marked with different lowercase superscript letters are significantly different *p* < 0.05; those marked with different uppercase superscript letters are highly significantly different *p* < 0.01; and values without a letter or sharing the same letter are not significantly different *p* > 0.05. 18L:6D: 18 h of light and 6 h of dark; 20L:4D: 20 h of light and 4 h of dark; 22L:2D: 22 h of light and 2 h of dark; 23L:1D: 23 h of light and 1 h of dark.

**Table 7:** Effects of different light cycles on skeletal parameters of yellow-feathered broiler chickens.

Items	Light Cycle				SEM	*p*-Value		
	18L:6D	20L:4D	22L:2D	23L:1D		Overall	Linear	Quadratic
Femur length (cm)	12.32 ^a^	12.24 ^a^	11.73 ^b^	11.96 ^ab^	0.07	0.013	0.006	0.782
Femur width (cm)	1.06	1.04	1.01	0.97	0.01	0.100	0.017	0.625
Tibia length (cm)	8.94 ^a^	8.96 ^ab^	8.55 ^ab^	8.65 ^b^	0.06	0.017	0.006	0.636
Tibia width (cm)	1.13 ^a^	1.13 ^a^	1.06 ^b^	1.08 ^ab^	0.01	0.040	0.012	0.863
Femur weight (g)	18.32	18.36	18.14	17.85	0.29	0.931	0.577	0.732
Tibia weight (g)	13.53	13.56	13.23	13.1	0.22	0.885	0.943	0.839
Tibia ash content (%)	51.74 ^ABab^	50.22 ^Bb^	52.23 ^Aa^	48.25 ^Cc^	0.38	<0.001	0.017	0.234
Tibia calcium content (%)	29.07	30.38	33.00	30.96	1.01	0.604	0.309	0.616
Tibia phosphorus content (%)	8.74	8.28	9.07	8.28	0.13	0.100	0.761	0.925

SEM: standard error of the mean. In the same row, values marked with different lowercase superscript letters are significantly different *p* < 0.05; those marked with different uppercase superscript letters are highly significantly different *p* < 0.01; and values without a letter or sharing the same letter are not significantly different *p* > 0.05. 18L:6D: 18 h of light and 6 h of dark; 20L:4D: 20 h of light and 4 h of dark; 22L:2D: 22 h of light and 2 h of dark; 23L:1D: 23 h of light and 1 h of dark.

**Table 8:** Effects of different light cycles on the tibia parameters of yellow-feathered broiler chickens.

Items	Light Cycle				SEM	*p*-Value		
	18L:6D	20L:4D	22L:2D	23L:1D		Overall	Linear	Quadratic
Bone volume (mm^3^)	150.68 ^a^	105.98 ^b^	113.85 ^b^	101.37 ^b^	6.82	0.011	0.004	0.084
Tissue volume (mm^3^)	929.71 ^a^	813.94 ^ab^	923.04 ^a^	705.59 ^b^	33.28	0.02	0.034	0.499
Bone volume fraction	0.17 ^a^	0.13 ^ab^	0.12 ^b^	0.14 ^ab^	0.01	0.046	0.048	0.036
Bone surface area (mm^2^)	1784.32	1509.89	1626.96	1371.47	76.35	0.288	0.133	0.862
Bone surface area to tissue volume ratio (mm^−1^)	1.91	1.88	1.76	1.93	0.07	0.825	0.826	0.599
Bone surface area to bone volume ratio (mm^−1^)	13.13	14.25	14.27	13.48	0.23	0.219	0.420	0.063
Trabecular number (mm^−1^)	0.58	0.66	0.57	0.68	0.03	0.458	0.551	0.951
Trabecular pattern factor (mm^−1^)	2.72	3.81	3.45	3.79	0.40	0.801	0.479	0.651
Structural pattern index	0.73	1.04	1.08	0.93	0.07	0.248	0.200	0.120
Trabecular connectivity	2580	3187	2756	2448.33	264.38	0.825	0.820	0.396
Trabecular connection density (mm^−3^)	2.77	4.06	2.97	3.51	0.39	0.711	0.777	0.524
Degree of anisotropy	0.56	0.53	0.49	0.49	0.02	0.748	0.303	0.935
Fractal dimension	2.49	2.47	2.44	2.45	0.01	0.592	0.238	0.767
Bone marrow cavity CT value	138.1	174.29	118.43	142.08	19.73	0.841	0.807	0.709
Bone mineral density (mg/cm^3^)	59.68	65.45	55.28	58.87	3.62	0.845	0.718	0.713
Bone mineral content (mg)	55.7	51.76	51.4	41.54	3.25	0.52	0.218	0.651
Trabecular CT value	1159.41	1210.04	1207.18	1131.01	23.44	0.634	0.831	0.248
Tissue mineral density (mg/cm^3^)	318.99	332.6	331.83	311.36	6.30	0.634	0.831	0.248
Tissue mineral content (mg)	44.39	35.21	37.84	31.48	2.40	0.304	0.120	0.724

SEM: standard error of the mean. In the same row, values marked with different lowercase superscript letters are significantly different *p* < 0.05; those marked with different uppercase superscript letters are highly significantly different *p* < 0.01; and values without a letter or sharing the same letter are not significantly different *p* > 0.05. 18L:6D: 18 h of light and 6 h of dark; 20L:4D: 20 h of light and 4 h of dark; 22L:2D: 22 h of light and 2 h of dark; 23L:1D: 23 h of light and 1 h of dark.

**Table 9:** The effect of different light cycles on the meat quality of yellow-feathered broilers.

Items	Light Cycle				SEM	*p*-Value		
	18L:6D	20L:4D	22L:2D	23L:1D		Overall	Linear	Quadratic
pH_45 min_	6.22 ^b^	6.35 ^a^	6.26 ^ab^	6.34 ^a^	0.02	0.032	0.127	0.303
pH_24 h_	5.72 ^Aa^	5.55 ^Cc^	5.75 ^Aa^	5.65 ^Bb^	0.01	<0.001	0.730	<0.001
L*_45 min_	45.73	45.99	45.92	46.27	0.21	0.850	0.452	0.931
a*_45 min_	2.38 ^Aa^	1.37 ^Bb^	1.38 ^Bb^	1.60 ^Bb^	0.10	<0.001	0.002	0.003
b*_45 min_	10.22 ^Aa^	9.88 ^ABab^	8.55 ^Cc^	9.32 ^Bb^	0.11	<0.001	<0.001	0.240
L*_24 h_	53.69 ^ab^	53.03 ^b^	54.13 ^ab^	54.8 ^a^	0.22	0.037	0.038	0.046
a*_24 h_	2.52	1.68	2.17	1.86	0.12	0.053	0.145	0.131
b*_24 h_	12.85	12.99	13.09	12.97	0.16	0.963	0.698	0.750
Shear force (N)	50.73 ^Bb^	51.09 ^Bb^	60.96 ^Aa^	43.33 ^Cc^	1.38	<0.001	0.773	0.013
Drip loss (%)	9.17 ^Aa^	8.64 ^Aa^	7.71 ^Aa^	4.37 ^Bb^	0.05	<0.001	<0.001	0.048
Cooking loss (%)	14.92 ^Aa^	12.00 ^ABab^	15.13 ^Aa^	11.30 ^Bb^	0.01	0.009	0.125	0.983

SEM: standard error of the mean. In the same row, values marked with different lowercase superscript letters are significantly different *p* < 0.05; those marked with different uppercase superscript letters are highly significantly different *p* < 0.01; and values without a letter or sharing the same letter are not significantly different *p* > 0.05. 18L:6D: 18 h of light and 6 h of dark; 20L:4D: 20 h of light and 4 h of dark; 22L:2D: 22 h of light and 2 h of dark; 23L:1D: 23 h of light and 1 h of dark.

**Table 10:** Effects of different light cycles on the nutritional components of yellow-feathered broiler chicken breast muscle: basis of dry matter (%).

Items	Light Cycle				SEM	*p*-Value		
	18L:6D	20L:4D	22L:2D	23L:1D		Overall	Linear	Quadratic
Moisture (%)	69.75	70.37	70.56	70.63	0.52	0.937	0.547	0.547
Crude protein (%)	75.50 ^BCbc^	77.41 ^ABab^	78.40 ^Aa^	74.12 ^Cc^	0.45	0.002	0.783	0.001
Crude fat (%)	14.9	12.83	12.39	12.95	0.40	0.118	0.351	0.932
Crude ash (%)	5.76 ^Aa^	5.51 ^Aa^	4.07 ^Bb^	4.88 ^ABab^	0.18	0.003	0.054	0.020

SEM: standard error of the mean. In the same row, values marked with different lowercase superscript letters are significantly different *p* < 0.05; those marked with different uppercase superscript letters are highly significantly different *p* < 0.01; and values without a letter or sharing the same letter are not significantly different *p* > 0.05. 18L:6D: 18 h of light and 6 h of dark; 20L:4D: 20 h of light and 4 h of dark; 22L:2D: 22 h of light and 2 h of dark; 23L:1D: 23 h of light and 1 h of dark.

**Table 11:** Effect of different lighting cycles on amino acid content in the breast muscle of yellow-feathered broilers (g/mg).

Items	Light Cycle				SEM	*p*-Value		
	18L:6D	20L:4D	22L:2D	23L:1D		Overall	Linear	Quadratic
Aspartic acid	29.16 ^Aa^	25.95 ^Aa^	20.74 ^Bb^	21.58 ^Bb^	0.92	<0.001	0.427	0.247
Threonine	14.00 ^Aa^	12.29 ^ABab^	10.04 ^Cc^	10.47 ^BCbc^	0.44	0.001	0.448	0.320
Serine	12.51 ^Aa^	10.81 ^ABab^	8.92 ^Cc^	9.29 ^BCbc^	0.39	<0.001	0.438	0.377
Glutamic acid	47.83 ^Aa^	40.83 ^Bb^	34.41 ^Bb^	35.99 ^Bb^	1.46	0.001	0.382	0.435
Proline	8.80 ^Aa^	8.65 ^Bb^	6.63 ^Bb^	6.69 ^Bb^	0.26	<0.001	0.149	0.073
Glycine	12.67 ^Aa^	10.82 ^Bb^	8.92 ^Cc^	9.43 ^BCbc^	0.40	<0.001	0.331	0.343
Alanine	17.67 ^Aa^	15.31 ^ABab^	12.60 ^Cc^	13.27 ^BCbc^	0.55	0.001	0.379	0.326
Cysteine	0.56 ^Bb^	0.91 ^Aa^	0.46 ^Bb^	0.51 ^Bb^	0.04	<0.001	<0.001	<0.001
Valine	14.35 ^Aa^	12.51 ^ABab^	10.44 ^Bb^	11.00 ^Bb^	0.44	0.002	0.393	0.347
Methionine	5.60	4.03	5.32	6.06	0.45	0.457	0.314	0.665
Isoleucine	13.68 ^Aa^	11.92 ^ABab^	9.97 ^Bb^	10.43 ^Bb^	0.42	0.002	0.443	0.381
Leucine	23.98 ^Aa^	20.67 ^ABab^	17.31 ^Bb^	18.08 ^Bb^	0.74	0.002	0.430	0.413
Tyrosine	9.08 ^Aa^	7.82 ^ABab^	6.36 ^Cc^	6.74 ^BCbc^	0.29	<0.001	0.325	0.285
Phenylalanine	9.90 ^Aa^	8.44 ^ABab^	6.73 ^Cc^	7.69 ^BCbc^	0.32	0.001	0.108	0.143
Histidine	18.80 ^Aa^	16.95 ^ABab^	14.25 ^Bb^	14.30 ^Bb^	0.58	0.005	0.740	0.488
Lysine	26.74 ^Aa^	23.2 ^ABab^	19.11 ^Cc^	20.28 ^Bb^	0.83	0.001	0.351	0.309
Arginine	17.58 ^Aa^	15.32 ^ABab^	12.52 ^Cc^	13.22 ^BCbc^	0.56	0.001	0.399	0.312

SEM: standard error of the mean. In the same row, values marked with different lowercase superscript letters are significantly different *p* < 0.05; those marked with different uppercase superscript letters are highly significantly different *p* < 0.01; and values without a letter or sharing the same letter are not significantly different *p* > 0.05. 18L:6D: 18 h of light and 6 h of dark; 20L:4D: 20 h of light and 4 h of dark; 22L:2D: 22 h of light and 2 h of dark; 23L:1D: 23 h of light and 1 h of dark.

## Data Availability

The raw data supporting the conclusions of this article will be made available by the authors upon request.

## Abbreviations

The following abbreviations are used in this manuscript:

BW	Body weight
ADFI	Average daily feed intake
ADG	Average daily gain
F/G	Feed to gain ratio
TP	Total protein
ALB	Albumin
GLB	Globulin
A/G	Albumin to globulin ratio
BUN	Blood urea nitrogen
ALT	Alanine aminotransferase
AST	Aspartate aminotransferase
ALP	Alkaline phosphatase
TBIL	Total bilirubin
GLU	Glucose
TGs	Triglycerides
T-CHO	Total cholesterol
HDL-C	High-density lipoprotein cholesterol
LDL-C	Low-density lipoprotein cholesterol
FL	Femur length
FW	Femur width
TL	Tibia length
TW	Tibia width
TWT	Tibia weight
FWT	Femur weight
BV	Bone volume
TV	Tissue volume
BV/TV	Bone volume fraction
BS	Bone surface area
BS/TV	Bone surface area to tissue volume ratio
BS/BV	Bone surface area to bone volume ratio
Tb. N	Trabecular number
Tb. Pf	Trabecular pattern factor
SMI	Structure model index
Connectivity	Trabecular connectivity
Conn. D	Connectivity density
DA	Degree of anisotropy
FD	Fractal dimension
Tb Cav. CT Value	CT value of the marrow cavity
BMD	Bone mineral density
BMC	Bone mineral content
Tb. CT Value	Trabecular bone CT value
TMD	Tissue mineral density
TMC	Tissue mineral content

## References

[1] Vida V., Szakály Z. (2023). Analysis of consumer behaviour in the European poultry meat market. Appl. Stud. Agribus. Commer..

[2] Roiter L., Vedenkina I., Roiter Y., Akopyan A., Eremeeva N. (2023). Niches in the market potential of poultry meat. E3S Web Conf..

[3] FAO (2025). Meat of Chickens, Fresh or Chilled.

[4] Zhang F., Chen Z., Shi J., Han C., Zhan Q., Ren Z., Yang X. (2025). Challenges and constraints to the sustainability of poultry farming in China. Anim. Biosci..

[5] Yang X., Cai B., Zhang Z., Mo Y., Zhou Z., Wu R., Kong S., Cai D., Zhang R., Li Z. (2024). Exploring variances in meat quality between Qingyuan partridge chicken and Cobb broiler: Insights from combined multi-omics analysis. Poult. Sci..

[6] Wu Y., Huang J., Quan S., Yang Y. (2022). Light regimen on health and growth of broilers: An update review. Poult. Sci..

[7] Jiang S., Fu Y., Cheng H. (2023). Daylight exposure and circadian clocks in broilers: Part I-photoperiod effect on broiler behavior, skeletal health, and fear response. Poult. Sci..

[8] Li C., Shu H., Gu X. (2025). Photoperiod management in farm animal husbandry: A review. Animals.

[9] Kim H.-J., Son J., Jeon J.-J., Kim H.-S., Yun Y.-S., Kang H.-K., Hong E.-C., Kim J.-H. (2022). Effects of photoperiod on the performance, blood profile, welfare parameters, and carcass characteristics in broiler chickens. Animals.

[10] Shynkaruk T., Buchynski K., Schwean-Lardner K. (2022). Lighting programme as a management tool for broilers raised without antibiotics—Impact on productivity and welfare. Br. Poult. Sci..

[11] Lalit K., Ameeta D.A., Kaajal N., Sourav B. (2025). A Review the Effect of Light on the Production of Broiler Chickens. J. Sci. Res. Rep..

[12] Brassó L.D., Komlósi I., Várszegi Z. (2025). Modern technologies for improving broiler production and welfare: A Review. Animals.

[13] Ghanima M.M.A., Abd El-Hack M.E., Abougabal M.S., Taha A.E., Tufarelli V., Laudadio V., Naiel M.A. (2021). Growth, carcass traits, immunity and oxidative status of broilers exposed to continuous or intermittent lighting programs. Anim. Biosci..

[14] Kwon B.Y., Park J., Kim D.H., Lee K.W. (2024). Assessment of welfare problems in broilers: Focus on musculoskeletal problems associated with their rapid growth. Animals.

[15] Zhang Y., Wang Z., Dong Y., Cao J., Chen Y. (2022). Effects of different monochromatic light combinations on Cecal microbiota composition and Cecal tonsil T lymphocyte proliferation. Front. Immunol..

[16] Yu M., Xu M., Wang G., Feng J., Zhang M. (2025). Effects of different photoperiods on melatonin level, cecal microbiota and breast muscle morphology of broiler chickens. Front. Microbiol..

[17] Tuell J.R., Park J.Y., Wang W., Cheng H.W., Kim Y.H.B. (2020). Functional/physicochemical properties and oxidative stability of ground meat from broilers reared under different photoperiods. Poult. Sci..

[18] Zhang H., Qi G., Wang K., Yang J., Shen Y., Yang X., Chen X., Yao X., Gu X., Qi L. (2023). Oxidative stress: Roles in skeletal muscle atrophy. Biochem. Pharmacol..

[19] Gratta F., Cabrol M.B., Xiccato G., Birolo M., Bordignon F., Trocino A. (2023). Effect of light restriction on productive results and behavior of broiler chickens. Poult. Sci..

[20] Tuell J.R., Park J.Y., Wang W., Cooper B., Sobreira T., Cheng H.W., Kim Y.H.B. (2020). Effects of photoperiod regime on meat quality, oxidative stability, and metabolites of postmortem broiler fillet (m. Pectoralis major) muscles. Foods.

[21] Freitas E.R., Costa H.S., Nepomuceno R.C., Silva L.P., Aguiar G.C., Lima P., Watanabe P.H. (2023). Bone Growth and Quality of Meat Quails Submitted to Different Lighting Programmes. Braz. J. Poult. Sci..

[22] van der Pol C.W., van Roovert-Reijrink I.A., Maatjens C.M., Gussekloo S.W., Kranenbarg S., Wijnen J., Pieters R.P., Schipper H., Kemp B., van den Brand H. (2019). Light-dark rhythms during incubation of broiler chicken embryos and their effects on embryonic and post hatch leg bone development. PLoS ONE.

[23] Guo Y., Tang H., Wang X., Li W., Wang Y., Yan F., Kang X., Li Z., Han R. (2019). Clinical assessment of growth performance, bone morphometry, bone quality, and serum indicators in broilers affected by valgus-varus deformity. Poult. Sci..

[24] (2020). Nutrient requirements of yellow-feathered broilers.

[25] (2010). Technical regulation for feeding and management of yellow-feathered broilers.

[26] (2004). Terminology and Measurement Methods for Poultry Performance.

[27] (2018). Determination of total phosphorus in feed—Spectrophotometric method.

[28] (2008). Meat and meat products—Determination of moisture content.

[29] (2025). National food safety standard—Determination of protein in foods.

[30] (2003). Determination of fat in foods.

[31] (2008). Inspection of germplasm for cultured fishes—Part 11: Determination of main amino acids in muscle.

[32] (2007). Determination of meat quality in livestock and poultry.

[33] Pal P., Dey D.K., Sharma B., Choudhary S.K., Sahu J., Kumar S., Ghosh S. (2019). Effect of light management in broiler production: A review. J. Entomol. Zool. Stud..

[34] Classen H.L., Annett C.B., Schwean-Lardner K.V., Gonda R., Derow D. (2004). The effects of lighting programmes with twelve hours of darkness per day provided in one, six or twelve hour intervals on the productivity and health of broiler chickens. Br. Poult. Sci..

[35] Downs K.M., Lien R.J., Hess J.B., Bilgili S.F., Dozier W.A. (2006). The effects of photoperiod length, light intensity, and feed energy on growth responses and meat yield of broilers. J. Appl. Poult. Res..

[36] Yang H., Xing H., Wang Z., Xia J., Wan Y., Hou B., Zhang J. (2015). Effects of intermittent lighting on broiler growth performance, slaughter performance, serum biochemical parameters and tibia parameters. Ital. J. Anim. Sci..

[37] Acosta-Rodriguez V.A., Rijo-Ferreira F., van Rosmalen L., Izumo M., Park N., Joseph C., Hepler C., Thorne A.K., Stubblefield J., Bass J. (2024). Misaligned feeding uncouples daily rhythms within brown adipose tissue and between peripheral clocks. Cell Rep..

[38] Yu M., Xu M., Wang G., Feng J., Zhang M. (2024). Effects of Different Photoperiods on Growth Performance, Glucose Metabolism, Acetylcholine, and Its Relative Acetylcholine Receptor Modulation in Broiler Chickens. Animals.

[39] Nazligue A., Türkyılmaz M.K., Karaarslan S., Kaya M. (2017). Effects of photoperiod length and light intensity on performance, carcass characteristics and heterophil to lymphocyte ratio in broilers. Kafkas Univ. Vet. Fak. Derg..

[40] Fidan E.D., Nazlıgül A., Türkyılmaz M.K., Aypak S.Ü., Kilimci F.S., Karaarslan S., Kaya M. (2017). Effect of photoperiod length and light intensity on some welfare criteria, carcass, and meat quality characteristics in broilers. Rev. Bras. Zootec..

[41] Lewis P., Danisman R., Gous R. (2009). Photoperiodic responses of broilers. I. Growth, feeding behaviour, breast meat yield, and testicular growth. Br. Poult. Sci..

[42] Schwean-Lardner K., Fancher B.I., Classen H.L. (2012). Impact of daylength on the productivity of two commercial broiler strains. Br. Poult. Sci..

[43] Shynkaruk T., Classen H.L., Crowe T.G., Schwean-Lardner K. (2019). The impact of dark exposure on broiler feeding behavior and weight of gastrointestinal tract segments and contents. Poult. Sci..

[44] Silva A.S., Jong I.C. (2019). The role of intermittent lighting schedules in broiler production systems. Livest. Sci..

[45] van der Sluis M., van der Eijk J.A.J., Garcia-Faria T.I., te Beest D.E., Wolthuis-Fillerup M., Jong I.C. (2025). Light spectrum and intensity preferences of fast- and slower-growing broilers vary by age, behaviour and time of day. Appl. Anim. Behav. Sci..

[46] Mosleh N., Nazifi S., Ghanadzadegan F. (2014). Effect of three different photoperiod schedules on serum leptin and lipid profile, abdominal fat pad adiposity and triglyceride content in broiler chickens. Bulg. J. Vet. Med..

[47] Zhang D., Zhao F., Wu X., Xi L., Wang X. (2020). Effects of Different Photoperiods on Feeding Behavior, Growth Performance and Blood Parameters of Broilers. Chin. J. Anim. Nutr..

[48] Saleh M.S., Al-Rubaie M.A.M.J., Khlati R.H. (2024). The Growth performance and some blood traits in broilers under different lighting regimens. Am. J. Biodivers..

[49] Fanatico A.C., Pillai P.B., Cavitt L.C., Owens C.M., Emmert J.L. (2005). Evaluation of Slower-Growing Broiler Genotypes Grown with and without Outdoor Access: Growth Performance and Carcass Yield. Poult. Sci..

[50] Lien R.J., Hess J.B., McKee S.R., Bilgili S.F., Townsend J.C. (2007). Effect of light intensity and photoperiod on live performance, heterophil-to-lymphocyte ratio, and processing yields of broilers. Poult. Sci..

[51] Baykalır Y., Şimşek U., Erışır M., Otlu O., Güngören G., Güngören A., Aslan S. (2020). Photoperiod effects on carcass traits, meat quality, and stress response in heart and lung of broilers. S. Afr. J. Anim. Sci..

[52] Rahimi G., Rezaei M., Hafezian H., Saiyahzadeh H. (2005). The effect of intermittent lighting schedule on broiler performance. Int. J. Poult. Sci..

[53] Khonitan D., Ariyadi B., Dono N.D. (2025). The Difference in Lighting Duration on Broiler Chicken Production and Carcass Quality. Proceedings of the 5th International Conference on Environmentally Sustainable Animal Industry (ICESAI 2024).

[54] Pap T.I., Szabó R.T., Bodnár Á., Pajor F., Egerszegi I., Podmaniczky B., Pacz M., Mezőszentgyörgyi D., Kovács-Weber M. (2024). Effect of lighting methods on the Production, behavior and meat quality parameters of broiler chickens. Animals.

[55] Perretti A., Oyeniran V.J., Cherry J.M., Whittle R.H., Grider Z., Nelson A.H., Kang S.W., Erf G.F., Weimer S.L. (2025). Effects of light wavelength on broiler performance, blood cell profiles, stress levels, and tibiotarsi morphology. Animals.

[56] Tekin M., Ünal N., Onbaşılar E.E. (2025). The effects of LED lights in different colors on fattening performance, litter characteristics, meat properties, and some welfare parameters in broilers. Ank. Univ. Vet. Fak. Derg..

[57] Alagawany M., ElNesr S.S., Farag M.R., El-Hack M.E.A., Khafaga A.F., Taha A.E., Tiwari R., Yatoo M.I., Bhatt P., Marappan G. (2019). Use of licorice (*Glycyrrhiza glabra*) herb as a feed additive in poultry: Current knowledge and prospects. Animals.

[58] Gordon J., Manley N.R. (2011). Mechanisms of thymus organogenesis and morphogenesis. Development.

[59] Ribatti D. (2015). The bursa of Fabricius. The Development of Immunologic Competence.

[60] Cheng Y., Guo B., Xu Y., Liu J., Yang W., Zhang Y., Zhang Y., Liu J., Zhu H., Luo G. (2025). Effects of monochromatic light on the development of immune organs, antioxidant capacity and immune response of thymus and bursa of Fabricius in Yangzhou geese. Animals.

[61] Mohamed R.A., Eltholth M.M., El-Saıdy N.R. (2014). Rearing broiler chickens under monochromatic blue light improve performance and reduce fear and stress during pre-slaughter handling and transportation. Biotechnol. Anim. Husb..

[62] Mench J., Weeks C.A., Butterworth A. (2004). Lameness. Measuring and Auditing Broiler Welfare.

[63] Cui Y., Wang J., Zhang H., Qi G., Wu S. (2021). Effect of photoperiod on eggshell quality and quality characteristics of tibia, femur, and ulna in laying ducks. Poult. Sci..

[64] Schwean-Lardner K., Fancher B.I., Gomis S., Van Kessel A., Dalal S., Classen H.L. (2013). Effect of day length on cause of mortality, leg health, and ocular health in broilers. Poult. Sci..

[65] Reis D., Torres R., Barbosa A., Euclydes R. (2011). Effects of lineage and sex on geometrical and biomechanical properties of broiler chickens tibias. Acta Sci.-Anim. Sci..

[66] Henry M., Pesti G. (2002). An Investigation of Calcium Citrate-Malate as a Calcium Source for Young Broiler Chicks. Poult. Sci..

[67] Park S., Birkhold S., Kubena L., Nisbet D., Ricke S. (2003). Effect of Storage Condition on Bone Breaking Strength and Bone Ash in Laying Hens at Different Stages in Production Cycles. Poult. Sci..

[68] Lopes T.S.B., Vasconcelos M.D.C., Costa B.T.A., Sousa L.S., Bertassoli B.M., Ocarino N.d.M., Serakides R., Lara L.J.C., Araújo I.C.S. (2024). Performance and bone health of broilers reared under artificial lighting and supplemented with different levels of vitamin D3. Rev. Bras. Zootec..

[69] Nasirzadeh N., Zamiri M.J., Akhlaghi A., Ghovvati S., Kargar S., Amini J. (2025). Influence of LED light spectra and photoperiods on performance, bone characteristics and related genes expression in broiler breeders. Br. Poult. Sci..

[70] Hanlon C., Zuidhof M.J., Rodriguez A., Takeshima K., Bédécarrats G.Y. (2023). Continuous exposure to red light induces photorefractoriness in broiler breeder pullets. Poult. Sci..

[71] Yang Y., Liu Q., Wang T., Pan J. (2020). Wavelength-specific artificial light disrupts molecular clock in avian species: A power-calibrated statistical approach. Environ. Pollut..

[72] Zuo Y., Hou Y., Wang Y., Yuan L., Cheng L., Zhang T. (2024). Circadian misalignment impairs oligodendrocyte myelination via Bmal1 overexpression leading to anxiety and depression-like behaviors. J. Pineal Res..

[73] Wei L., Chen W., Huang L., Wang H., Su Y., Liang J., Lian H., Xu J., Zhao J., Liu Q. (2022). Alpinetin ameliorates bone loss in LPS-induced inflammation osteolysis via ROS mediated P38/PI3K signaling pathway. Pharmacol. Res..

[74] Kim M.J., Parvin R., Mushtaq M.M.H., Hwangbo J., Kim J.H., Na J.C., Kim D.W., Kang H.K., Kim C.D., Cho K.O. (2013). Influence of monochromatic light on quality traits, nutritional, fatty acid, and amino acid profiles of broiler chicken meat. Poult. Sci..

[75] Li W., Guo Y., Chen J., Wang R., He Y., Su D. (2010). Influence of lighting schedule and nutrient density in broiler chickens: Effect on growth performance, carcass traits and meat quality. Asian-Australas. J. Anim. Sci..

[76] Gündoğar U.C., Onbaşılar E.E., Ahlat O. (2024). Effects of abrupt and gradual light/dark switching on growth performance, behavior, villus development, meat characteristics, and immunity of broilers. Anim. Sci. J..

[77] Chromý V., Vinklárková B., Šprongl L., Bittová M. (2015). The Kjeldahl method as a primary reference procedure for total protein in certified reference materials used in clinical chemistry. I. A review of Kjeldahl methods adopted by laboratory medicine. Crit. Rev. Anal. Chem..

[78] Dickinson J.M., Rasmussen B.B. (2013). Amino acid transporters in the regulation of human skeletal muscle protein metabolism. Curr. Opin. Clin. Nutr. Metab. Care.

[79] Wilhelm A.E., Maganhini M.B., Hernández-Blazquez F.J., Ida E.I., Shimokomaki M. (2010). Protease activity and the ultrastructure of broiler chicken PSE (pale, soft, exudative) meat. Food Chem..

[80] Alnahhas N., Le Bihan-Duval E., Baéza E., Chabault M., Chartrin P., Bordeau T., Cailleau-Audouin E., Meteau K., Berri C. (2015). Impact of divergent selection for ultimate pH of pectoralis major muscle on biochemical, histological, and sensorial attributes of broiler meat. J. Anim. Sci..

[81] Akyüz H.Ç., Onbaşılar E.E. (2023). Carcass, visceral organ, and meat quality properties of two broiler hybrids differing in growth rates. Anim. Sci. J..

[82] Bennato F., Ianni A., Martino C., Grotta L., Martino G. (2021). Evaluation of chemical composition and meat quality of breast muscle in broilers reared under light-emitting diode. Animals.

[83] Kruk Z.A., Yun H., Rutley D.L., Lee E.J., Kim Y.J., Jo C. (2011). The effect of high pressure on microbial population, meat quality and sensory characteristics of chicken breast fillet. Food Control.

[84] Lindahl G., Lundström K., Tornberg E. (2001). Contribution of pigment content, myoglobin forms and internal reflectance to the colour of pork loin and ham from pure breed pigs. Meat Sci..

[85] Mancini R.A., Hunt M.C. (2005). Current research in meat color. Meat Sci..

[86] Mir N.A., Rafiq A., Kumar F., Singh V., Shukla V. (2017). Determinants of broiler chicken meat quality and factors affecting them: A review. J. Food Sci. Technol..

[87] Chartrin P., Meteau K., Juin H., Bernadet M.D., Guy G., Larzul C., Rémignon H., Mourot J., Duclos M.J., Baéza E. (2006). Effects of intramuscular fat levels on sensory characteristics of duck breast meat. Poult. Sci..

[88] Liu M.Q. (2024). Effects of Photoperiod on Sexual Maturity and Its Mechanism in Chahua Chicken No. 2. Ph.D. Dissertation.

[89] Zhang L., Hao Z., Zhao C., Zhang Y., Li J., Sun B., Tang Y., Yao M. (2021). Taste compounds, affecting factors, and methods used to evaluate chicken soup: A review. Food Sci. Nutr..

[90] Ordóñez-Araque R., Rodríguez-Villacres J., Urresto-Villegas J. (2020). Electronic nose, tongue and eye: Their usefulness for the food industry. Vitae.

[91] Surányi J., Zaukuu J.L.Z., Friedrich L., Kovács Z., Horváth F., Németh C., Kókai Z. (2021). Electronic tongue as a correlative technique for modeling cattle meat quality and classification of breeds. Foods.

[92] de Sousa Fontes V.M., de Sousa Galvão M., de Carvalho L.M., do Nascimento Guedes F.L., dos Santos Lima M., Bezerra T.K.A., Madruga M.S. (2024). Thiamine, cysteine and xylose added to the Maillard reaction of goat protein hydrolysate potentiates the formation of meat flavoring compounds. Food Chem..

[93] Ding Y., Zhou T., Liao Y., Lin H., Deng S., Zhang B. (2022). Comparative studies on the physicochemical and volatile flavour properties of traditional deep fried and circulating-air fried hairtail (*Trichiurus lepturus*). Foods.

[94] Li J., Li Z., Ran J., Yang C., Lin Z., Liu Y. (2022). LC/MS-based lipidomics to characterize breed-specific and tissue-specific lipid composition of chicken meat and abdominal fat. LWT.

